# Transcriptomic screening of novel targets of sericin in human hepatocellular carcinoma cells

**DOI:** 10.1038/s41598-024-56179-y

**Published:** 2024-03-05

**Authors:** Jiraporn Jantaravinid, Napatara Tirawanchai, Sumate Ampawong, Kanchana Kengkoom, Anchaleekorn Somkasetrin, Vorthunju Nakhonsri, Pornanong Aramwit

**Affiliations:** 1 Center of Excellence in Bioactive Resources for Innovative Clinical Applications, Department of Pharmacy Practice, Faculty of Pharmaceutical Sciences, Chulalongkorn University, 254 Phayathai Road, Pathumwan, Bangkok, 10330 Thailand; 2 Department of Biochemistry, Faculty of Medicine Siriraj Hospital, Mahidol University, 2, Wanglang Road, Bangkoknoi, Bangkok, 10700 Thailand; 3 Department of Tropical Pathology, Faculty of Tropical Medicine, Mahidol University, 420/6, Ratchawithi Road, Ratchathewi, Bangkok, 10400 Thailand; 4 Research and Academic Support Office, National Laboratory Animal Center, Mahidol University, 999, Salaya, Puttamonthon, Nakorn Pathom, 73170 Thailand; 5 National Biobank of Thailand (NBT), National Science and Technology Development Agency (NSTDA), 144 Innovation Cluster 2 Building (INC) Tower A, Thailand Science Park, Khlong Nueng, Khlong Luang District, Pathum Thani, 12120 Thailand; 6 The Academy of Science, The Royal Society of Thailand, Dusit, Bangkok, 10330 Thailand

**Keywords:** Sericin, Transcriptome profiling, Hepatoprotection, Target identification, Gene ontology, High-throughput screening, Pharmacogenetics

## Abstract

Sericin, a natural protein derived from *Bombyx mori,* is known to ameliorate liver tissue damage; however, its molecular mechanism remains unclear. Herein, we aimed to identify the possible novel targets of sericin in hepatocytes and related cellular pathways*.* RNA sequencing analysis indicated that a low dose of sericin resulted in 18 differentially expressed genes (DEGs) being upregulated and 68 DEGs being downregulated, while 61 DEGs were upregulated and 265 DEGs were downregulated in response to a high dose of sericin (FDR ≤ 0.05, fold change > 1.50). Functional analysis revealed that a low dose of sericin regulated pathways associated with the complement and coagulation cascade, metallothionine, and histone demethylate (HDMs), whereas a high dose of sericin was associated with pathways involved in lipid metabolism, mitogen-activated protein kinase (MAPK) signaling and autophagy. The gene network analysis highlighted twelve genes, *A2M, SERPINA5, MT2A, MT1G, MT1E, ARID5B, POU2F1, APOB, TRAF6, HSPA8, FGFR1,* and *OGT,* as novel targets of sericin. Network analysis of transcription factor activity revealed that sericin affects NFE2L2, TFAP2C, STAT1, GATA3, CREB1 and CEBPA. Additionally, the protective effects of sericin depended on the counterregulation of *APOB, POU2F1, OGT, TRAF6*, and *HSPA5*. These findings suggest that sericin exerts hepatoprotective effects through diverse pathways at different doses, providing novel potential targets for the treatment of liver diseases.

## Introduction

The liver is a vital organ in the human body. They are organized as a structurally diverse group of specialized tissues that are involved in crucial biochemical functions. It can be broadly defined as metabolic, exocrine, and endocrine in nature. It also regulates functions involving detoxification, hormone production, decomposition of red blood cells, protein anabolism and nutrient metabolism^1^. Hepatotoxicity is defined as liver injury caused by chemical or drug abuse, excessive alcohol consumption, infections, or autoimmune disorders that results in impaired liver function^2–4^. Despite tremendous strides in modern medicine, there are few available drugs that effectively restore liver function, exert hepatoprotective effects or promote the regeneration of hepatic cells. However, natural extracts are gaining increasing attention as pharmacotherapeutic agents for the treatment of liver diseases due to their improved efficacy and safety compared to the currently used drugs. The use of natural medicine for the treatment of liver diseases has a long history, and their therapeutic effects are achieved by targeting multiple factors and pathways. Several studies have shown that natural extracts protect against CCl_4_-related hepatotoxicity by improving antioxidant enzyme activity and suppressing lipid peroxidation^5,6^.

Sericin is a natural protein produced by the silkworm *Bombyx mori (B. mori),* a holometabolous insect belonging to the Bombycidae family in the Lepidoptera order. *B. mori* produces sericin and, together with fibroin, creates silk thread to form cocoons^7^. Due to its variety of biological properties, including antioxidation, anti-inflammatory and anticancer properties, sericin has been extensively used as a medical drug^8^. It has been used to treat and prevent liver injury, diabetes, cardiovascular diseases, skin diseases, and neurological diseases^9–12^. Sericin is also known to interact with several molecular targets underlying its pharmacological effects against diseases^8^. You-Gui Li et al. revealed that sericin protects against lipid peroxidation in membranous organelles by upregulating superoxide dismutase (SOD), catalase (CAT), and plasma glutathione peroxidase (GSH-PX) enzymes in the liver^13^. Chengjun Song et al. showed that sericin was able to increase the signal transduction effect of insulin by increasing the expression levels of key factors involved in insulin resistance (IR), such as insulin receptor substrate-1 (IRS-1), phosphatidylinositol-3-kinase (PI3K) and AKT, in the liver insulin-PI3K/AKT signaling pathway, thereby promoting the synthesis of hepatic glycogen^14^. Ampawong et al. demonstrated that sericin balances pancreatic cell biosynthesis by enhancing lipid dysregulation in hypercholesterolemia, resulting in a reduction in dysmorphic mitochondria, particularly in the heart and liver^15^. Heng-Da Wang et al. suggested that sericin effectively decreases inflammatory infiltration, restores impaired liver cells, and repairs liver damage^16^. Although sericin has been reported to ameliorate pathological damage in liver tissue and has a hepatoprotective effect against liver damage, systematic investigations of the molecular mechanisms by which sericin prevents liver diseases are incomplete.

Next-generation sequencing-based transcriptome profiling is a powerful approach for interpreting the molecular mechanisms underlying biological processes^17^ and has been widely applied in drug discovery and development to identify drug-associated genes^18^. Using RNA sequencing (RNA-seq) and gene expression analysis, this study aimed to identify candidate genes and promising targets of sericin in order to generate a novel hypothesis on the molecular mechanism of action of sericin in relation to various human liver diseases, including viral hepatitis, liver cirrhosis, and hepatocellular carcinoma. The results of the Gene Ontology (GO), Kyoto Encyclopedia of Genes and Genomes (KEGG), and Reactome database analyses, the protein‒protein interaction network analysis (PPIN) and the transcription factor activity analysis, highlighted several biological processes modulated by sericin. This transcriptome analysis provides new insight into the regulatory and beneficial effects of sericin on hepatic cells.

## Results

### Assessment of the cytotoxic effect of sericin on HepG2 cells

**Figure 1:**
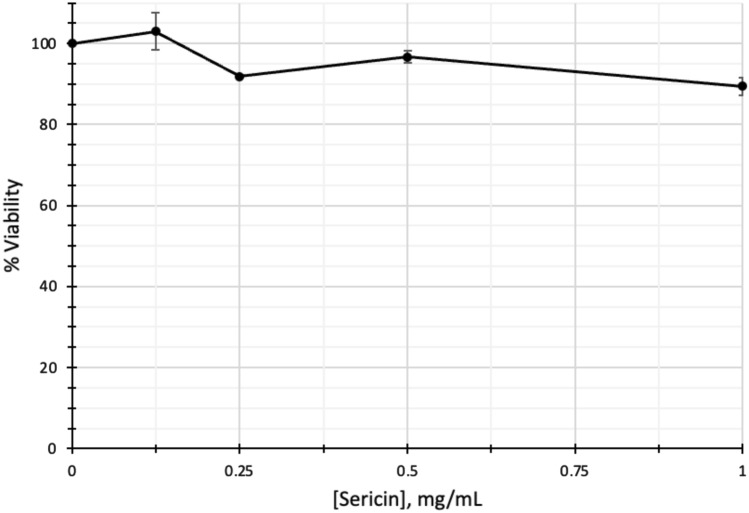
Cytotoxic effect of sericin on HepG2 cells.

The cytotoxic effect of sericin was assessed in HepG2 cells using an MTT assay. The concentrations of sericin used in this study were 0, 0.125, 0.25, 0.5, and 1 mg/mL. The results showed no cytotoxicity following sericin administration (Fig. 1). Hence, we selected a low concentration (0.125 mg/mL) and a high concentration (1 mg/mL) of sericin for the subsequent RNA-seq analysis.

### Identification of differentially expressed genes (DEGs)

**Figure 2:**
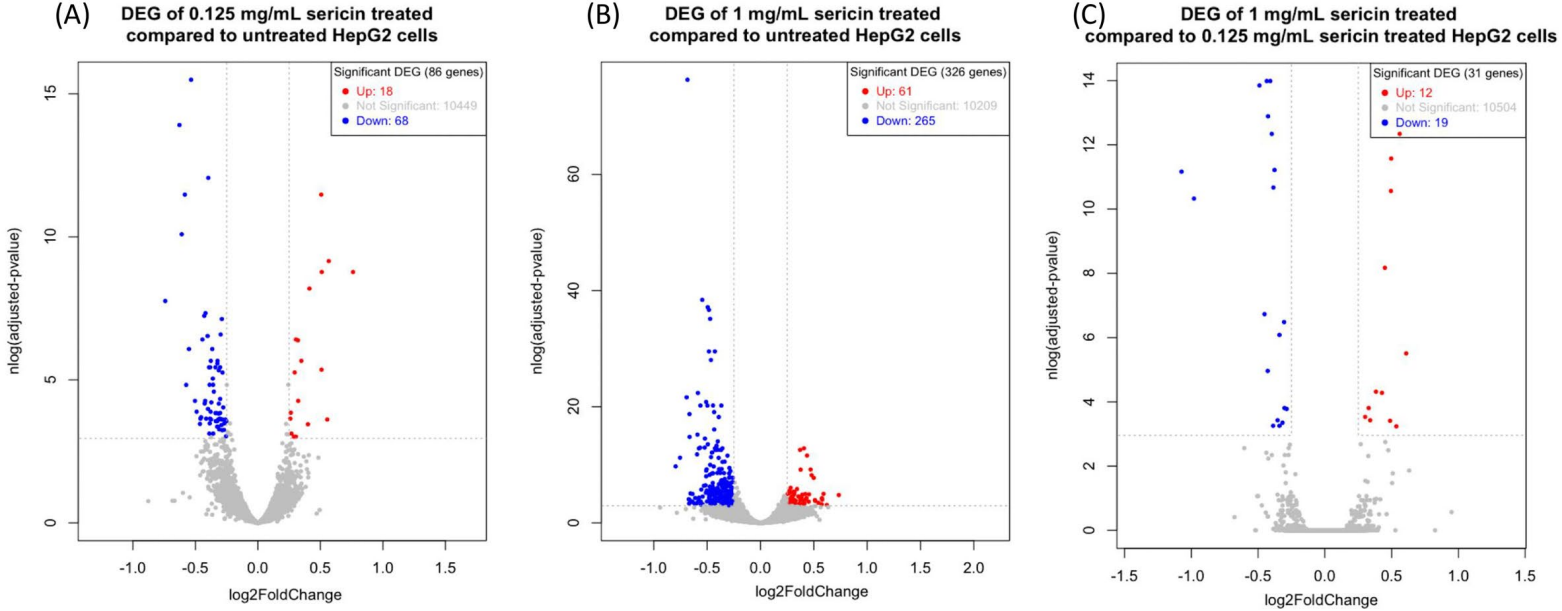
Genes that are differentially expressed after sericin administration (blue and red represent downregulated and upregulated genes, respectively). Volcano plots illustrating the magnitude and significance of DEGs between the (**A**) 0.125 mg/mL sericin-treated group vs. untreated group, (**B**) 1 mg/mL sericin-treated group vs. untreated group, and (**C**) 0.125 mg/mL sericin-treated group vs. 1 mg/mL sericin-treated group.

After the hepatic cells were treated with low (0.125 mg/mL) or high (1 mg/mL) concentrations of sericin, hierarchical clustering was performed to establish the impact of different concentrations of sericin on the transcriptome. Hierarchical clustering was performed for the untreated sericin or control (n = 3), 0.125 mg/mL sericin-treated (n = 3) and 1 mg/mL sericin-treated (n = 3) groups. The transcriptome of each group was normally segregated based on the sericin-treated group (Supplementary Fig. S1). A total of 60,606 transcripts were identified. Of the 86 DEGs, 18 were upregulated, whereas 68 were downregulated in the 0.125 mg/mL sericin group compared with the untreated group. The volcano plots showed that the number of DEGs between the 1 mg/mL sericin-treated group and the untreated group differed from that between the 0.125 mg/mL sericin-treated group and the untreated group. Three hundred twenty-six (326) genes exhibited significant alterations in gene expression. Among them, 61 DEGs were upregulated and 265 DEGs were downregulated. Interestingly, 31 DEGs were found between the 0.125 mg/mL treatment group and the 1 mg/mL treatment group. There were 12 upregulated genes and 19 downregulated genes (padj ≤ 0.05, fold change > 1.5) (Table 1, Fig. 2, and Supplementary Table S1).

**Table 1:** Numbers of differentially expressed genes (DEGs) in the 0.125 mg/mL sericin treatment group vs. untreated, 1 mg/mL sericin treatment vs. untreated, and 0.125 mg/mL sericin treatment vs. 1 mg/mL.

	Total number of DEGs (Fold change ratio > 1.5)	Expression pattern	
		Upregulated	Downregulated
0.125 mg/mL vs. untreated	86	18	68
1 mg/mL vs. untreated	326	61	265
0.125 mg/mL vs. 1 mg/mL	31	12	19

**Figure 3:**
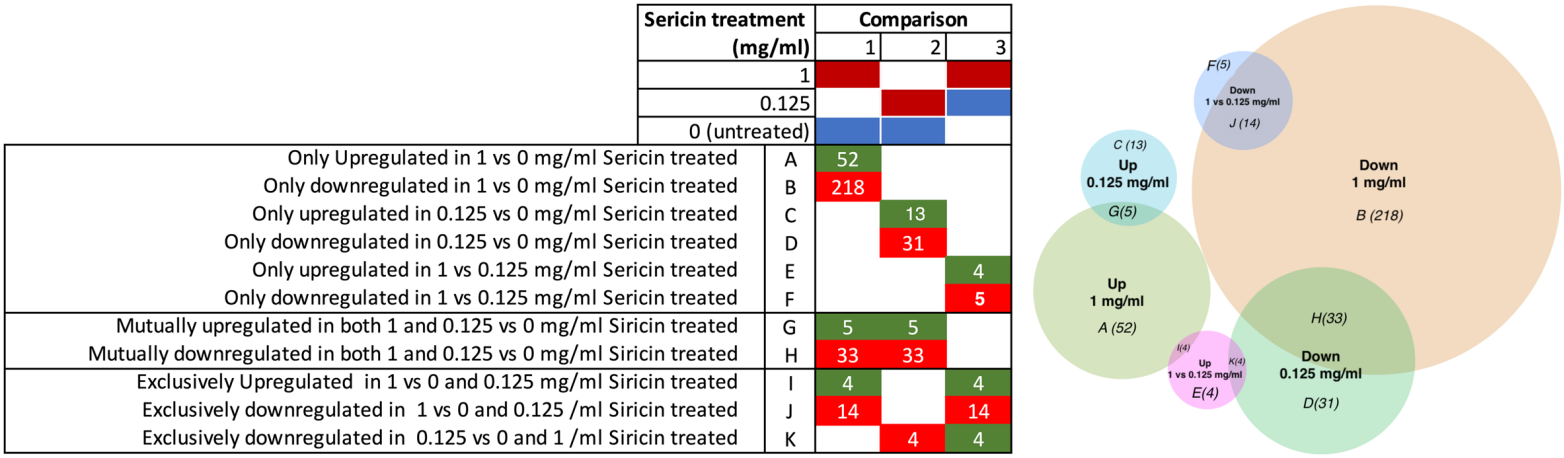
Venn diagram showing the shared DEGs identified by pairwise analyses.

Furthermore, the shared DEGs associated with sericin administration were analyzed via a Venn diagram (Fig. 3). The 0.125 mg/mL sericin-treated group and the 1 mg/mL sericin-treated group shared 5 upregulated and 33 downregulated genes. In contrast, there were 4 unique upregulated genes and 14 unique downregulated genes between the 0.125 mg/mL group and the 1 mg/mL group. These genes, as demonstrated by our findings, are strongly associated with hepatic protection.

### Functional enrichment analysis

#### GO analysis

GO analysis was performed using the hypergeometric distribution to functionally annotate the DEGs. GO terms were analyzed for both the 0.125 mg/mL sericin group and the untreated and 1 mg/mL sericin group and untreated groups. The 86 DEGs identified in the 0.125 mg/mL sericin treatment group vs. the untreated group were enriched in 42 GO biological process terms. (Supplementary Table S2). The top three significantly enriched GO terms were positive regulation of T-helper 1 cell cytokine production, positive regulation of transcription from RNA polymerase II promoter in response to hypoxia and negative regulation of smooth muscle cell migration. On the contrary, GO terms. In contrast, GO term analysis of the 326 DEGs identified in the 1 mg/mL sericin group revealed 90 enriched GO terms (Supplementary Table S3). The top three considerably enriched GO terms were nuclear speck, negative regulation of transcription from DNA template, and RNA polymerase II cis-regulatory region sequence-specific DNA binding. Interestingly, GO analysis of the DEGs between the 0.125 mg/mL and 1 mg/mL groups revealed that these DEGs were enriched in protein refolding, G protein-coupled receptor binding and heat shock proteins (Supplementary Table S4).

### KEGG and reactome pathway analysis

**Figure 4:**
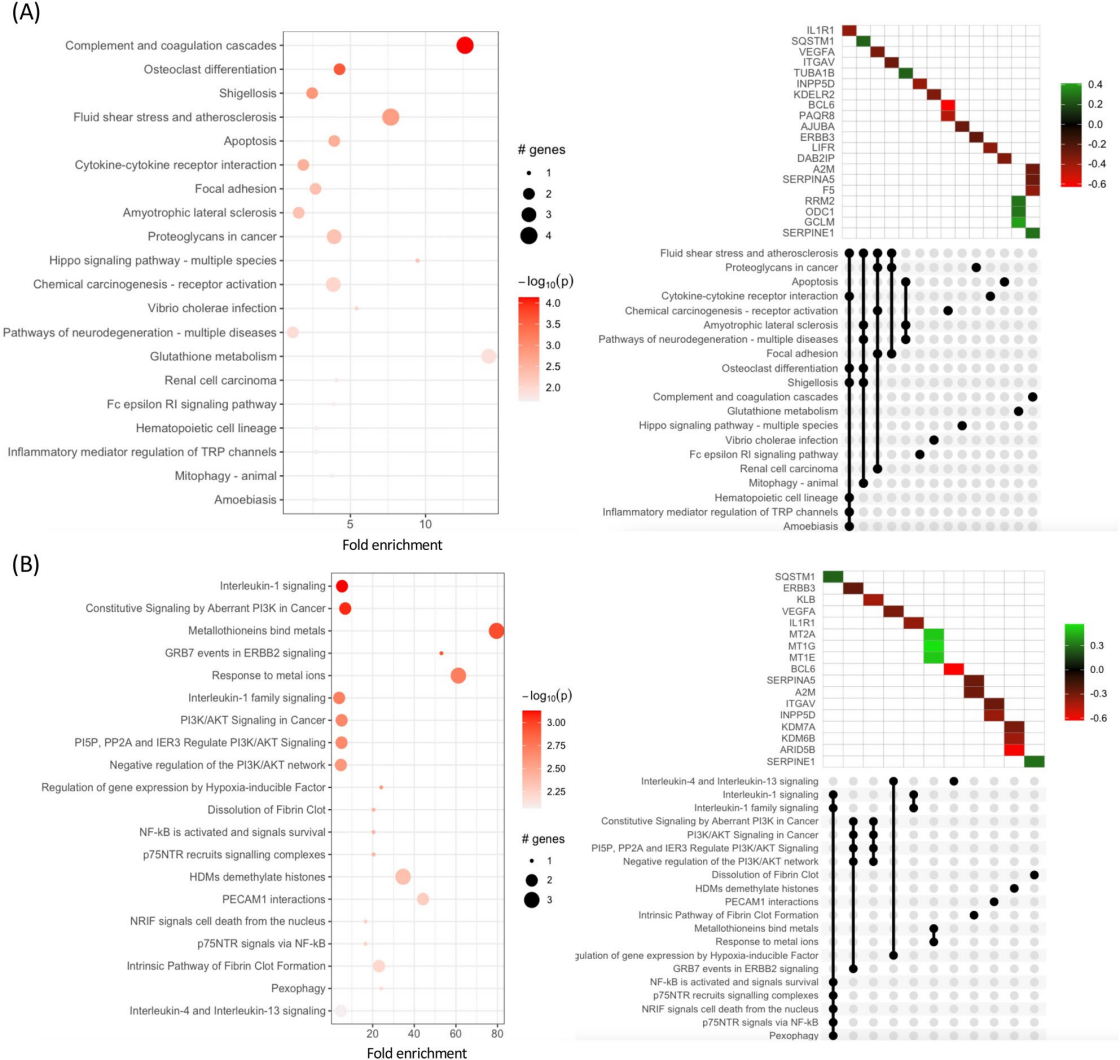
Pathway enrichment analysis of DEGs between the 0.125 mg/mL treatment group and the untreated group. (**A**) KEGG pathway enrichment. (**B**) Reactome pathway enrichment: the y-axis indicates the pathway name. The x-axis indicates the enrichment factor in each pathway. The bubble size indicates the number of genes. The color bar indicates the corrected p-value; orange represents a higher value, and light orange represents a lower value. The UpSet plot below indicates the interactions of the sets. The X-axis indicates the intersections of enriched terms. The main plot shows a heatmap of genes at corresponding intersections, colored depending on the up- or downregulation value. The UpSet plot below indicates the interactions of the sets. The X-axis indicates the UpSet plot of intersections of enriched terms. The main plot shows a heatmap of genes at corresponding intersections, colored by up- or downregulation.

The biological interpretation of the DEGs was further achieved using the KEGG and Reactome databases. KEGG pathway enrichment and Reactome analyses were performed to identify the functions of the DEGs. In 0.125 mg/mL sericin vs. untreated group, the significant DEGs (padj < 0.01, fold change > 1.5) were mapped to the KEGG and Reactome databases (Fig. 4, and Supplementary Table S5). The four significantly enriched KEGG pathways involved complement and coagulation cascades, glutathione metabolism, atherosclerosis, and apoptosis. Among them, the upregulation of members of the complement and coagulation cascades pathway *(A2M, SERINA5)* was observed only in the low-dose group. In addition, the two novels significantly enriched Reactome pathways included the intrinsic pathway of fibrin clot formation *(A2M, SERPINA5),* metallothionein-binding metals, and response to metal ions *(MT2A, MT1G, MT1E).* Interestingly, the presence of metallothionein-binding metals and the response to metal ions were correlated with glutathione metabolism, which was found in the KEGG analysis.

**Figure 5:**
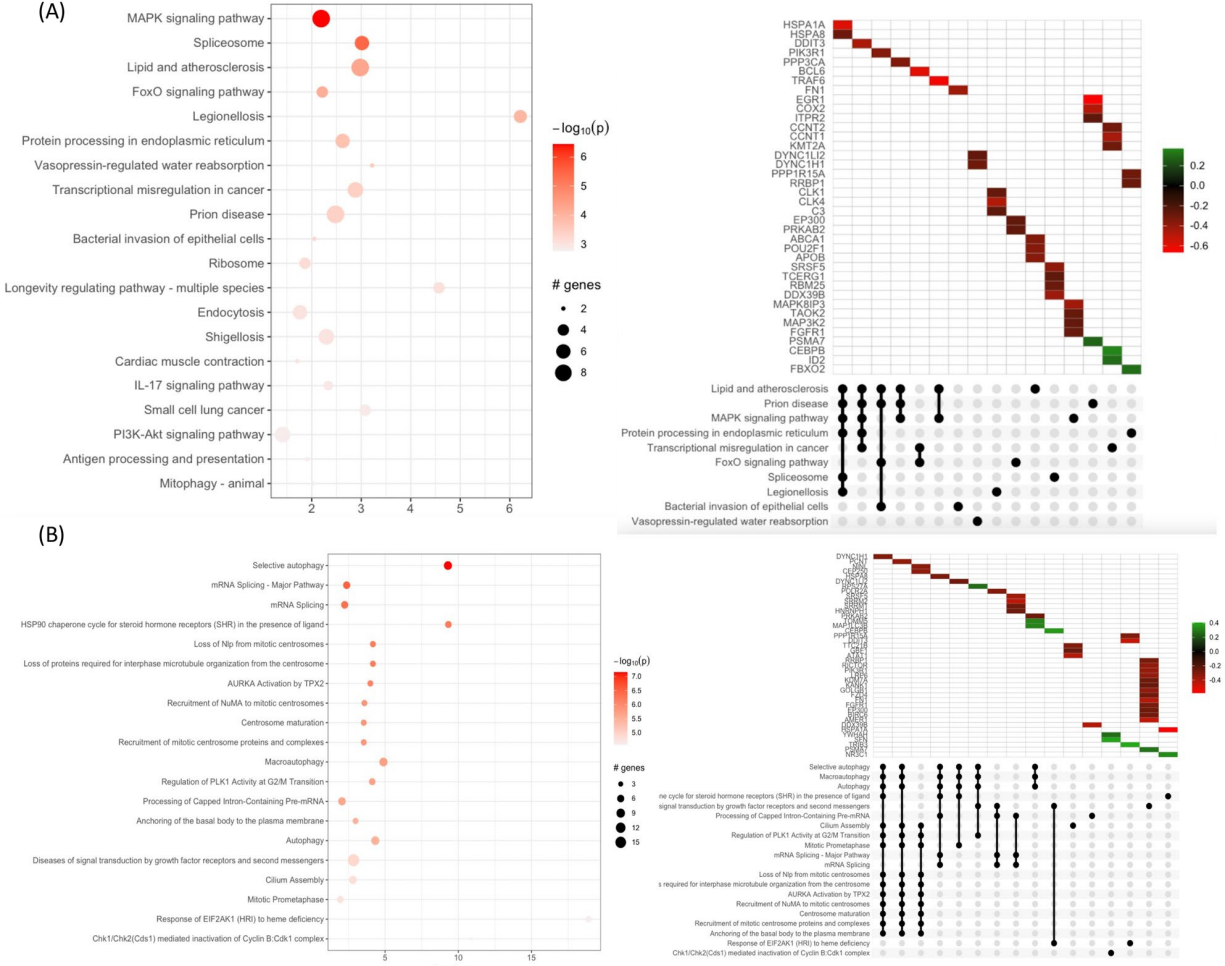
Pathway enrichment analysis of DEGs between the 1 mg/mL treatment group and the untreated group. (**A**) KEGG pathway enrichment. (**B**) Reactome pathway enrichment: the y-axis indicates the pathway name. The x-axis indicates the enrichment factor in each pathway. The bubble size indicates the number of genes. The color bar indicates the corrected p-value; orange represents a higher value, and light orange represents a lower value. The UpSet plot below indicates the interactions of the sets. The X-axis indicates the intersections of enriched terms. The main plot shows a heatmap of genes at corresponding intersections, colored depending on the up- or downregulation values.

Among the 1 mg/mL sericin-treated vs. untreated strains, the two most enriched KEGG pathways included the MAPK signaling pathway *(TRAF6, HSPA8)* and lipid and atherosclerosis *(POU2F1, APOB)* pathways*,* whereas the four most enriched Reactome pathways involved selective autophagy, macroautophagy, autophagy *(MAP1LC3B, DYNC1H1, HSPA5),* and signal transduction by growth factor receptors and second messengers. Among the significantly enriched Reactome pathways, selective autophagy, microautophagy, and autophagy were regulated upon sericin administration. This observation suggested that sericin may have a modulatory impact on hepatocellular carcinoma cells. (Fig. 5, and Supplementary Table S6).

**Figure 6:**
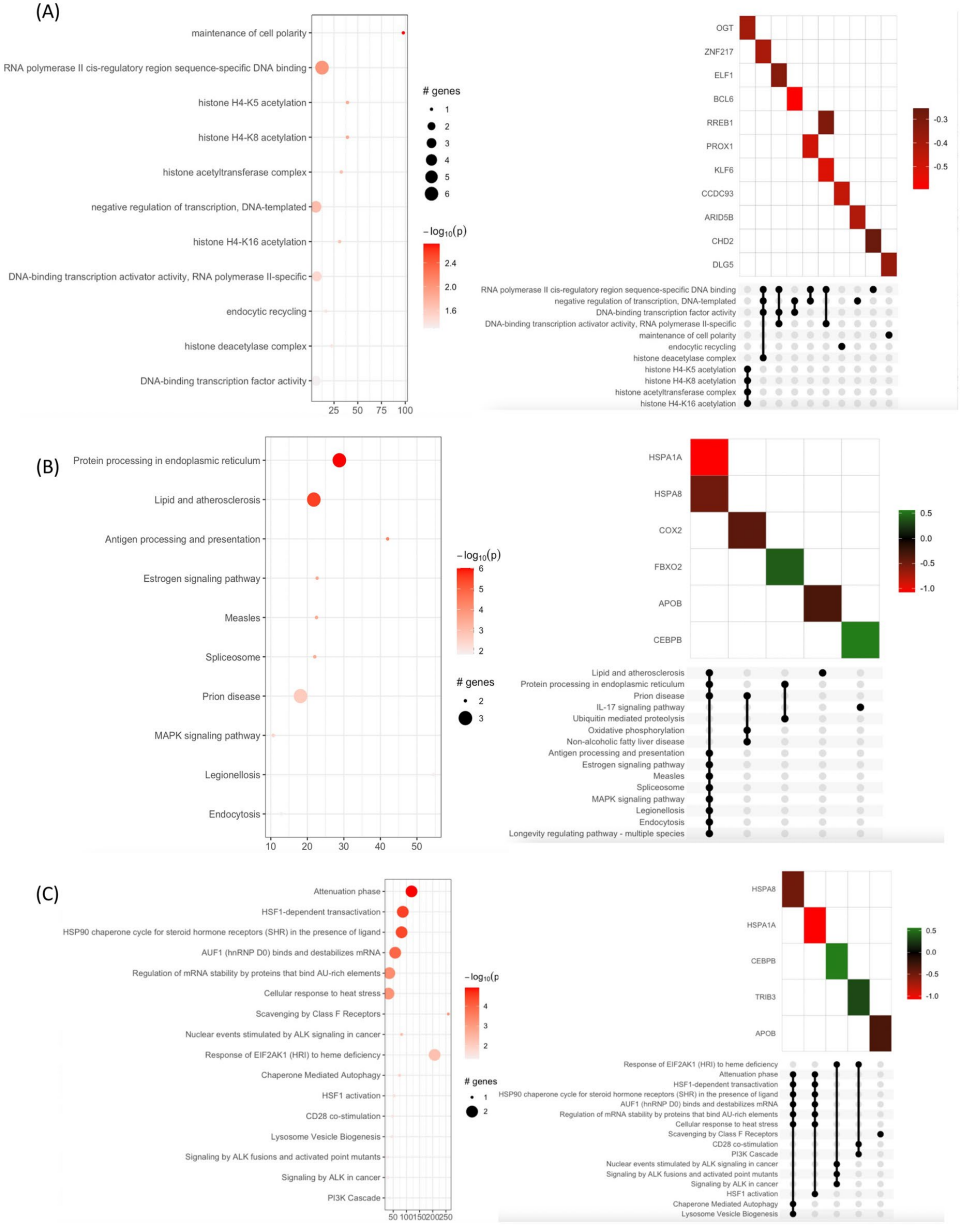
Pathway enrichment analysis of common DEGs between 0.125 mg/mL and 1 mg/mL sericin vs. untreated sericin. (**A**) KEGG pathway enrichment analysis of the common DEGs between the 0.125 mg/mL and 1 mg/mL groups. (**B**) KEGG and (**C**) Reactome pathway enrichment analysis for the contrast DEGs in the same comparisons. The y-axis indicates the pathway name. The x-axis indicates the enrichment factor in each pathway. The bubble size indicates the number of genes. The color bar indicates the corrected p-value; orange represents a higher value, and light orange represents a lower value. The UpSet plot below indicates the interactions of the sets. The X-axis indicates the intersections of enriched terms. The main plot shows a heatmap of genes at corresponding intersections, colored depending on the up- or downregulation values.

The results for the common differentially expressed genes (DEGs) in both the 0.125 mg/mL treatment group and the untreated and 1 mg/mL treatment groups compared with the untreated sericin treatment group revealed that histone modification was a crucial pathway (Fig. 6A). The O-GlcNAc transferase (OGT) gene was identified as a key player in several significantly enriched pathways among these common DEGs. This pattern suggested a threshold effect in which sericin at both tested concentrations triggered a uniform cellular response involving *OGT*, indicating an inherent protective mechanism. The consistent presence of *OGT* among the DEGs of both the low-dose and high-dose sericin groups underscores its role in activating important cellular pathways, thereby highlighting the potential of sericin for effective hepatoprotection. KEGG and Reactome enrichment analyses were performed on the *HSPA8* and *HSPA1A* genes, which were differentially expressed between the 0.125 mg/mL and 1 mg/mL sericin treatment groups. These genes were notably involved in pathways such as HSF-dependent transactivation and the HSP90 chaperone cycle (Fig. 6B,C, and Supplementary Table S7). This discovery emphasizes the crucial role of sericin dosage in modulating stress response pathways and highlights its dose-specific influence on hepatoprotective mechanisms.

## ,

### Protein–protein interaction network (PPIN) analysis

**Figure 7:**
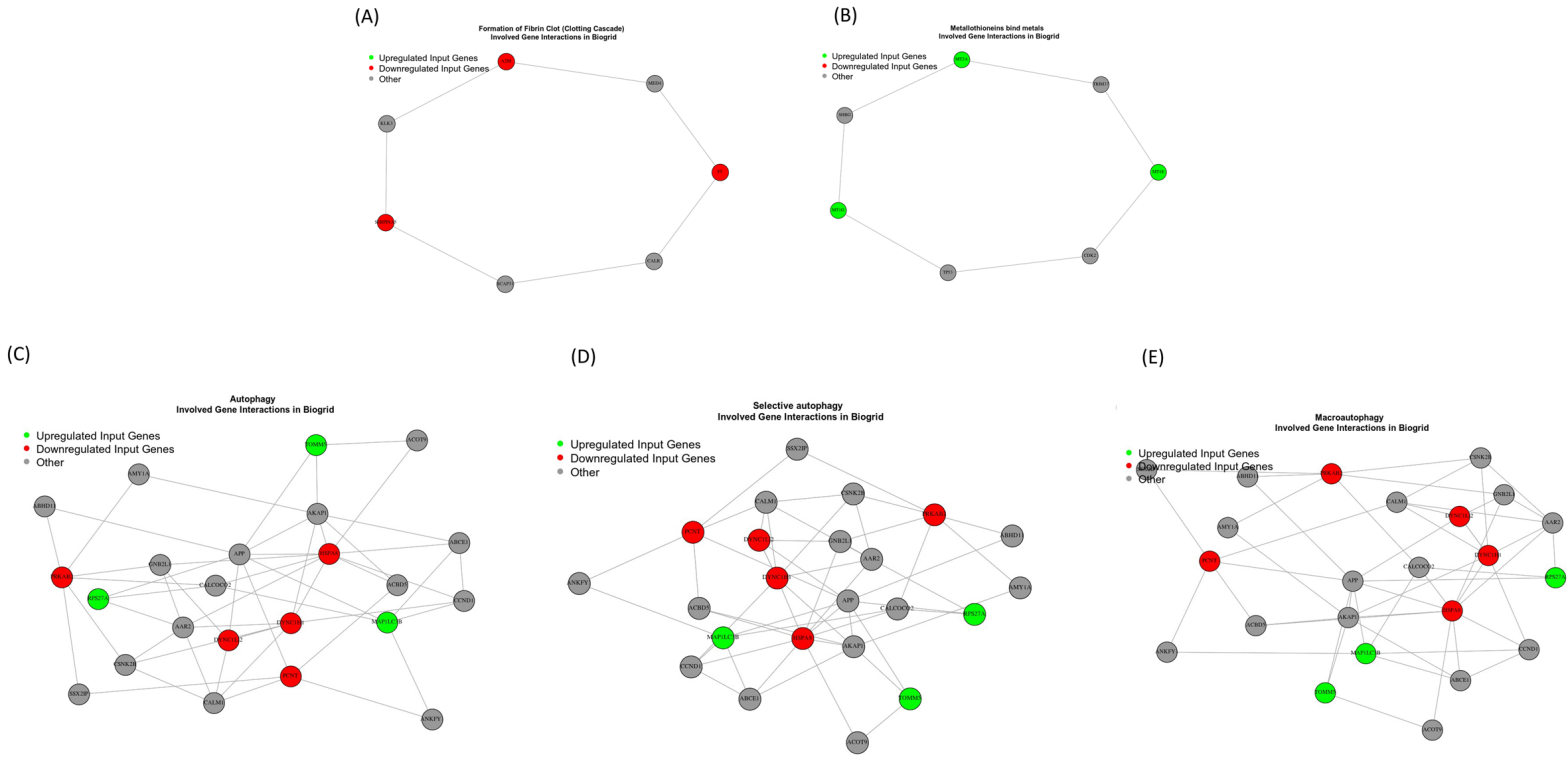
The critical protein complex affected by sericin treatment according to the PPI network. (**A**) Formation of fibrin clots. (**B**) Metallothionines bind metals. (**C**) Autophagy. (**D**) Selective autophagy. (**E**) Macroautophagy. Red indicates downregulation, and green indicates upregulation.

To explore the potential interactions at the protein level, DEGs were mapped in Biogrid, and a PPI network was constructed for DEGs in different groups at a stringent cutoff. The PPI network showed that the DEGs between the 0.125 mg/mL and untreated groups, the 1 mg/mL and untreated groups and the 0.125 mg/mL and 1 mg/mL groups, as well as the related functions and pathways identified by the GO, KEGG, and Reactome analyses, were correlated. Interestingly, all genes in the network are involved in the intrinsic pathway of fibrin clot formation and metallothionine binding metals, with autophagy emerging as a novel network aspect (Fig. 7).

### Expression of key genes involved in the hepatoprotective mechanisms of sericin

The differential expression of key genes associated with sericin administration was identified via KEGG, Reactome, and PPIN pathway analyses (Figs. 3, 4, 5, 6, 7). The key genes associated with the interesting and novel pathways are highlighted. At 0.125 mg/mL sericin vs. untreated, there were seven upregulated genes (*RRM2, ODC1, GCLM, SERPINE1, MT2A, MT1G, MT1E*) related to glutathione metabolism and complement, coagulation cascades, metallothionine-binding metals and the response to metal ions. In contrast, there were five downregulated DEGs *(BCL6, PAQR8*, *A2M, SERPINA5, F5)* related to chemical carcinogenesis-receptor activation, complement and coagulation cascades. Interestingly, *A2M* and *SERPINA5* were found to be shared genes according to the KEGG and Reactome enrichment analyses. At 1 mg/mL sericin vs. untreated, there were 17 downregulated DEGs *(HSPA1A, HSPA8, DDIT3, PIK3R1, PPP3CA, TRAF6, ABCA1, POU2F1, APOB, MAPK8IP3, TAOK2, MAP3K2, FGFR1, DYNC1H1, HSPA5, PRKAB2*, *DCNT)* that are were involved in lipid metabolism and atherosclerosis, the MAPK signaling pathway, selective autophagy, microautophagy and autophagy in hepatic cells, while 4 genes *(MAP1LC3B, MAP1LC3B, RRS27A, TOMM5)* associated with autophagy were upregulated. Furthermore, key genes identified via GO analysis are listed in Supplementary Tables S2 and S3.

### Identification of transcription factors

Transcription factors play vital roles in several gene regulatory mechanisms. The transcription factors associated with the DEGs were identified to further investigate the regulatory mechanisms of sericin in HepG2 cells. To define TF activity as a marker of therapeutic response, dcoupleR was used to statistically analyze gene expression and map the genes to the DoRothEA regulon gene set. The total activity score generated by dcoupleR represented the degree to which TFs regulated the gene expression network.

**Figure 8:**
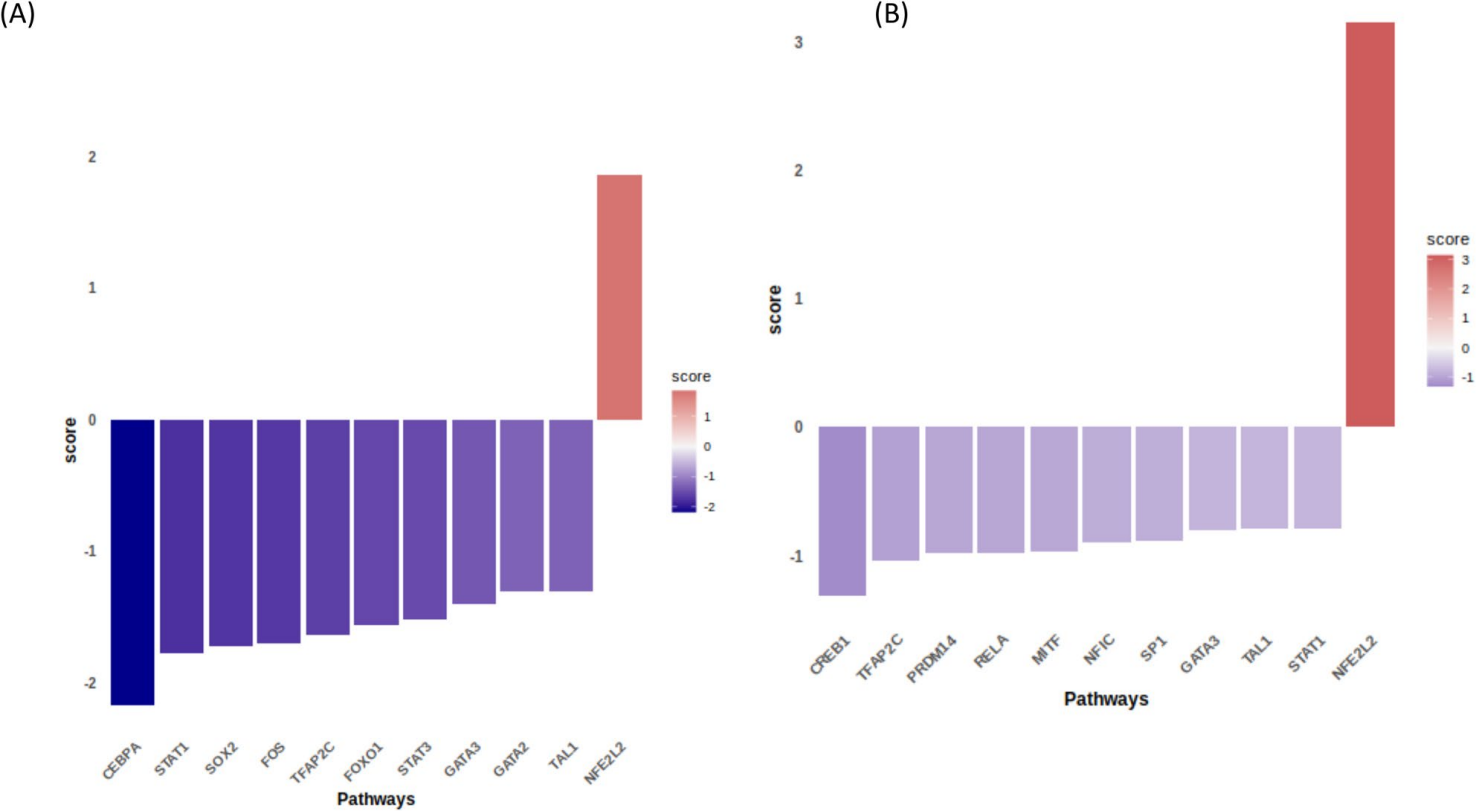
Box plot visualizing the changes in transcription factor activity upon sericin administration. (**A**) Transcription factor activity at 0.125 mg/mL. (**B**) Transcription factor activity at 1 mg/mL. The X-axis indicates the transcription factor pathway. The y-axis indicates the transcription factor score. The purple box indicates deactivation. The pink box indicates activation.

A bar plot of the changes in transcription factor (TF) activity upon treatment with 0.125 mg/mL sericin was constructed, which revealed eleven significant TFs. One TF, NFE2L2 (NFE2-like bZIP transcription factor 2), was activated and the following TFs were deactivated: CEBPA (CCAAT enhancer binding protein alpha), STAT1 (signal transducer and activator of transcription 1), SOX2 (SRY-box transcription factor 2), FOS (fos proto-oncogene or AP-1 transcription factor subunit), TFAP2C (transcription factor AP-2 gamma), FOXO1 (forkhead Box O1), STAT3 (signal transducer and activator of transcription 3), GATA3 (GATA binding protein 3), GATA2 (GATA binding protein 2) and TAL1 (TAL bHLH transcription factor 1) (Fig. 8). Notably, the activity of hepatic NFE2L2, a master regulator of the oxidative response, was increased upon low-dose sericin treatment. Investigating individual genes within the NFE2L2 TF regulon revealed the greatest increase in expression upon low dose sericin treatment in *IL1R1, SORBS1, RREB1, GCLM, TXNRD1,* and *SQSTM1.* These genes, which respond to detoxifying and antioxidant challenges in oxidative stress, exhibit cytoprotective effects, thereby preventing hepatic inflammation and injury by counteracting cytotoxicity. (Supplementary Fig. S2). In contrast, the most significant TF that exhibited a decrease in TF activity upon low-dose sericin treatment was CEBPA, which is involved in cellular differentiation, growth, and energy metabolism. Activation of CEBPA was indicated by decreased expression of the *ARIDB5, FILPP1L, NFIL3, ZBTB38, IL1R1, NEDD9, TGFBR3, MMD, PTCHD4, KANK1* and *PAQR8* genes as well as overexpression of *MRPL3*, which ameliorated liver injury and inflammation (Supplementary Fig. S2). These analyses revealed that CEBPA, NFE2L2 and their downstream target genes are important modulators of hepatoprotection upon low-dose sericin treatment. In addition, the significant changes in the expression of the TFs inferred from their regulon at 1 mg/mL were associated with the active TF NFE2L2 and the deactivated TFs CREB1 (cAMP responsive element binding protein 1), TFAP2 (transcription factor AP-2 gamma), PRDM14 (PR/SET domain 14), RELA (RELA proto-oncogene or NF-kB subunit), MITF (melanocyte inducing transcription factor), NFIC (nuclear Factor I C), SP1 (Sp1 transcription factor), GATA3 (GATA binding protein 3), TAL1 (AL bHLH transcription Factor 1), and STAT1 (signal transducer and activator of transcription 1) (Fig. 8). Notably, the network of NFE2L2 TF regulon *(PLEC, RREB1, CHD9, TXNRD1, GSTQ, GCLM, TGIF1 and NCOA7)* exhibited the highest increase of NFE2L2 TF activity (Supplementary Fig. S3). Deactivation of TF CREB1 has been described in fibrogenesis, and its activity was considerably decreased upon high-dose sericin treatment. The activity of CREB1, as inferred from changes in the expression of its regulon, involved alterations in *FN1, EGR1, FAT1, URTN, CHD9,* and *RREB1* (Supplementary Fig. S3). Interestingly, four pioneer TFs, STAT1, TFAP2C, GATA3 and NFE2L2, were affected by sericin treatment. Among these TFs, NFE2L2 was the activated, while STAT1, TFAP2C and GATA3*,* involved in inflammatory and lipid metabolism*,* were the deactivated upon sericin treatment.

### Effect of sericin on acetaminophen-induced hepatotoxicity

**Figure 9:**
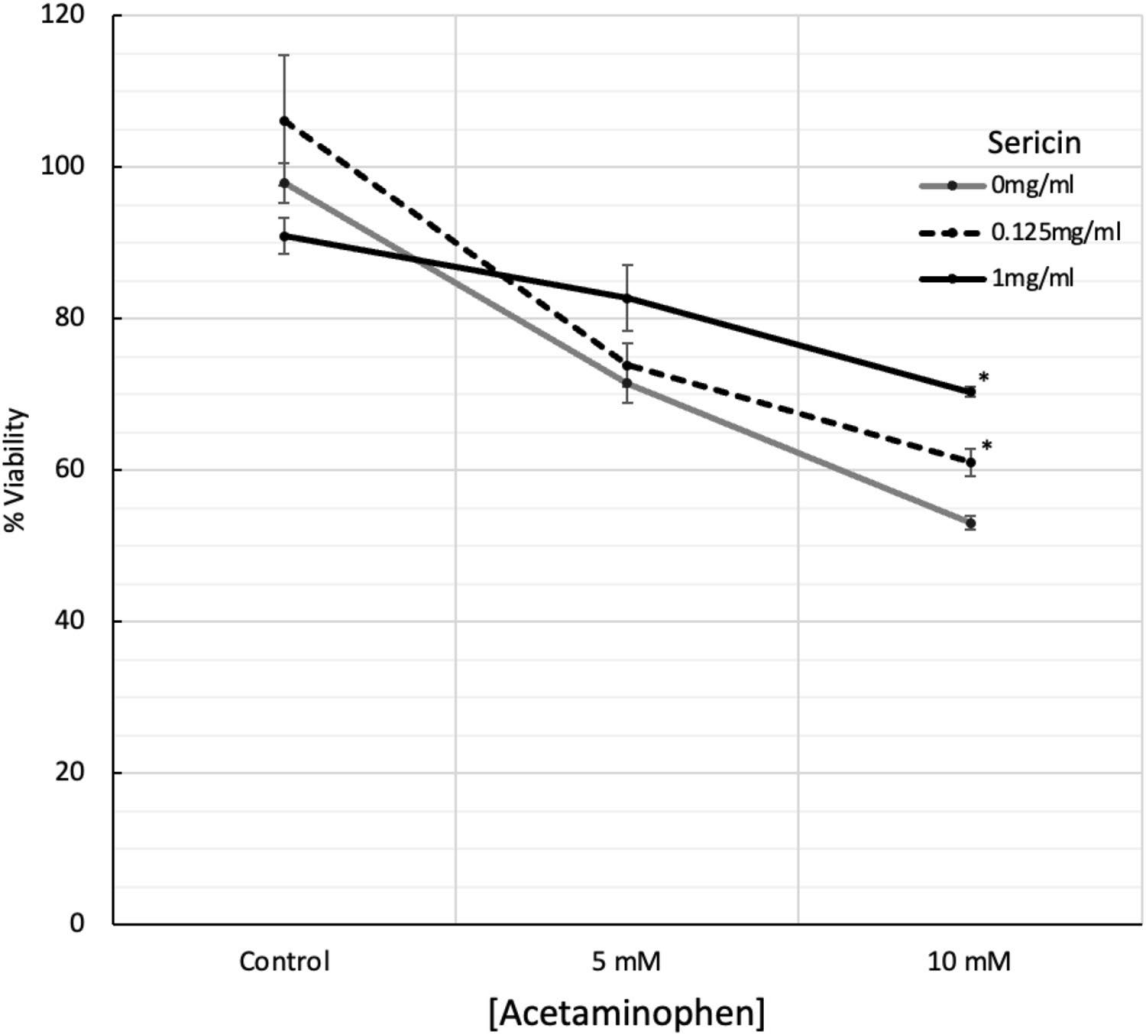
Cell viability in acetaminophen-induced hepatotoxicity treated with sericin. Data are expressed as means ± SEM. Statistical analysis was performed using a paired t-test. The asterisk (*) indicates a statistically significant difference (p < 0.05) between the sericin-treated groups (0.125 and 1 mg/mL) and the untreated control group.

The cytotoxicity of acetaminophen (APAP) was assessed in HepG2 cells via MTT assay. Cell viability decreased with increasing APAP concentrations (2.5–40 mM), showing more than 30% and 40% reductions at 5 mM and 10 mM, respectively (Supplementary Fig. S4). The IC50 was 4.2 mM. Co-treatment with APAP and sericin mitigated APAP-induced cell death in a dose-dependent manner (Fig. 9). Both high and low concentrations of sericin significantly reduced cell death in the presence of 10 mM APAP but were less effective in the presence of 5 mM APAP. As a result, 5 mM and 10 mM APAP were selected for further studies on the hepatoprotective effects of sericin.

**Figure 10:**
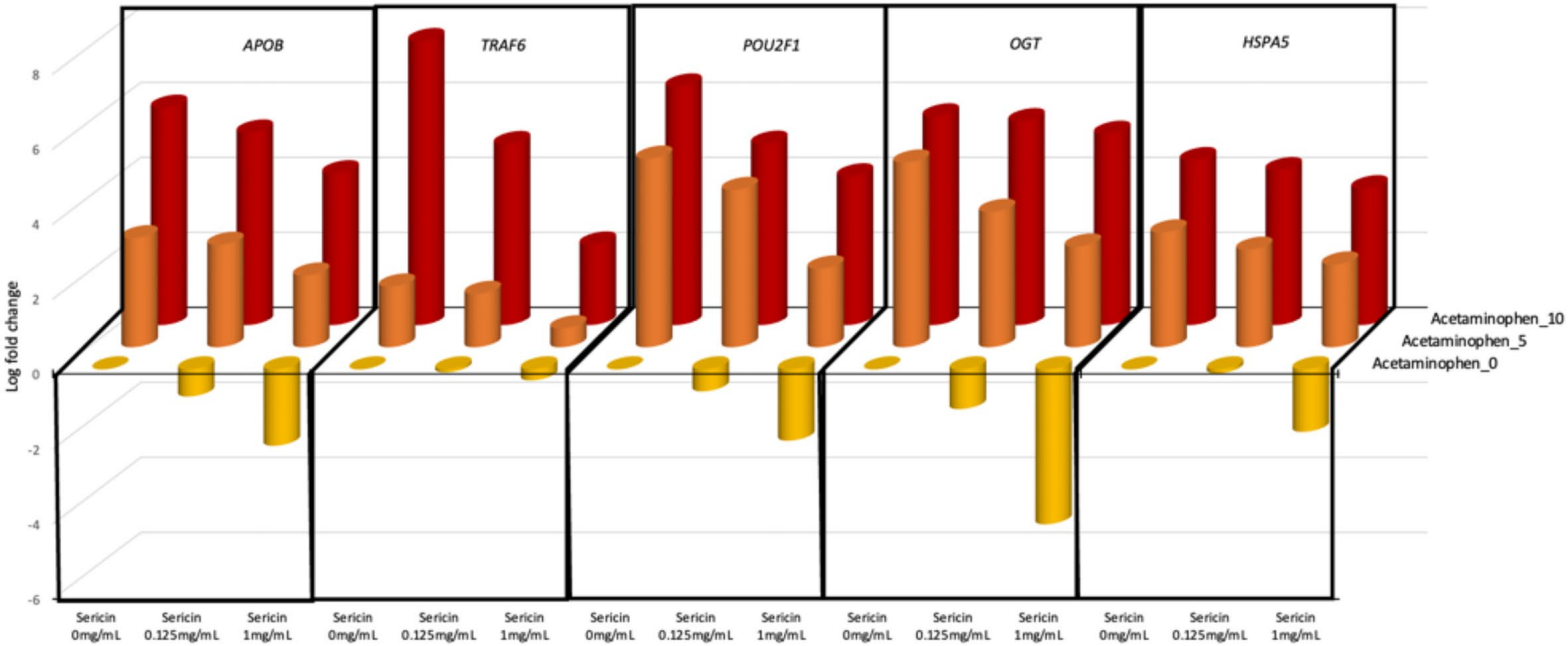
Expression of genes associated with sericin-mediated hepatoprotection in response to hepatotoxicity induced by acetaminophen.

To validate the hepatoprotective effects of sericin, gene expression assays were conducted in the presence of varying concentrations of APAP and sericin. Multiple regression analysis revealed that *APOB, TRAF6, POU2F1, OGT,* and *HSPA5* expression correlated significantly with both treatments (Table 2 and Fig. 10), underscoring the potential role of sericin in counteracting APAP-induced hepatotoxicity via gene modulation. Additionally, the changes in *TRAF6* expression in response to different treatments provide insights into the protective effects of sericin against APAP-induced hepatotoxicity. After treatment with 0.125 mg/ml sericin, *TRAF6* expression did not change, indicating a lack of direct effect at this concentration. However, when hepatotoxicity was induced using APAP, the same concentration of sericin led to a notable alteration in *TRAF6* expression. Intriguingly, a higher sericin dose of 1 mg/mL upregulated *TRAF6* but not to the same extent. These findings suggested that while sericin can modulate gene expression in a dose-dependent manner, its hepatoprotective effects become more pronounced under stress conditions, such as those induced by APAP.

**Table 2:** Multiple regression analysis of the hepatoprotective effect of sericin against acetaminophen-induced liver injury.

Gene	Acetaminophen				Sericin				Overall-P	Adj. R-squared
	Estimate	SE	t-value	p-value	Estimate	SE	t-value	p-value		
*APOB*	0.5647	0.0199	28.3074	**3.28E − 18**	− 1.3572	0.1793	− 7.5694	**1.98E − 07**	**1.66E − 17**	0.9724
*TRAF6*	0.5560	0.0814	6.8276	**2.17E − 06**	− 2.4580	0.6405	− 3.8374	**0.0012**	**1.59E − 06**	0.7479
*POU2F1*	0.5594	0.0747	7.4870	**2.34E − 07**	− 2.0304	0.6716	− 3.0234	**0.0065**	**7.36E − 07**	0.7146
*OGT*	0.6255	0.0585	10.6954	**5.89E − 10**	− 1.4732	0.5257	− 2.8023	**0.0107**	**2.86E − 09**	0.8318
*HSPA5*	0.4372	0.0374	11.6902	**1.18E − 10**	− 0.7750	0.3361	− 2.3055	**0.0314**	**6.31E − 10**	0.8543
*DYNC1H1*	0.5634	0.0763	7.3876	**2.88E − 07**	− 1.1102	0.6855	− 1.6195	0.1203	**1.40E − 06**	0.6966
*A2M*	0.7235	0.1272	5.6873	**1.21E − 05**	− 1.3144	1.1434	− 1.1496	0.2633	**5.53E − 05**	0.5694
*MAP1LC3B*	− 0.1169	0.0467	− 2.5039	**0.0206**	0.4435	0.4196	1.0568	0.3026	0.0541	0.1704
*MT1G*	− 0.5987	0.1379	− 4.3421	**0.0003**	0.8226	1.2395	0.6637	0.5141	**1.20E − 03**	0.4229
*MT1E*	− 0.3053	0.0610	− 5.0023	**5.96E − 05**	0.2781	0.5487	0.5068	0.6176	**2.60E − 04**	0.5011
*MT2A*	− 0.3646	0.0488	− 7.4748	**2.40E − 07**	0.1744	0.4384	0.3979	0.6947	**1.11E − 06**	0.7031
*SERPINA5*	0.4820	0.0675	7.1425	**4.82E − 07**	− 0.2387	0.6066	− 0.3935	0.6979	**2.22E − 06**	0.6829
*HSPA8*	0.6118	0.0761	8.0387	**7.62E − 08**	− 0.1525	0.6840	− 0.2230	0.8257	**3.42E − 07**	0.7347

Significant values are in bold.

### Verification of transcriptomic data by RT‒PCR

**Figure 11:**
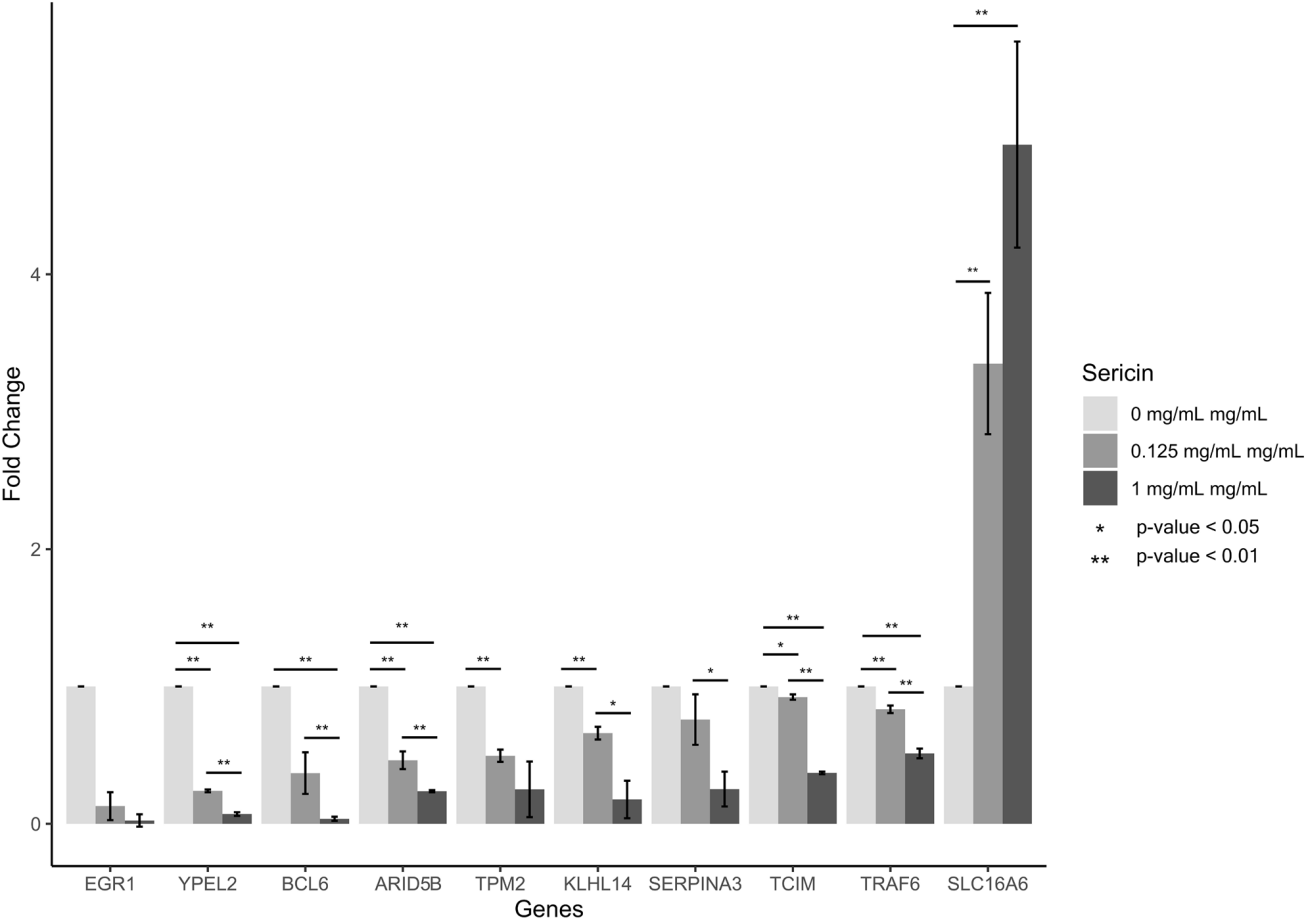
RT‒PCR illustrating the changes in the expression of the selected genes in HepG2 cells treated with 0.125 mg/mL sericin vs. untreated and 1 mg/mL sericin vs. untreated. The PCR products were normalized to GAPDH and are expressed as the fold change. The data are expressed as the mean ± SEM.

To further evaluate the accuracy of the RNA-Seq transcriptome data, ten candidate DEGs with a stringent cutoff (p < 0.01) were selected from both the 0.125 mg/mL sericin vs. untreated and 1 mg/mL sericin vs. untreated groups. This selection included upregulated genes, such as *SLC16A;* downregulated genes, namely *ARID5, BCL6, EGR1, KLHL14, SERPINA3, TCIM, TPM2, TRAF6, and YPEL2,* all of which are involved in different functional pathways. The expression levels of the selected DEGs were measured using qRT-PCR. Results exhibited the expression of *SLC16A6* was upregulated and expression of *ARID5, BCL6, EGR1, KLHL14, SERPINA3, TCIM, TPM2, TRAF6, and YPEL2* were downregulated in a dose-dependent manner (Fig. 11, Supplementary Table S8). To ensure the reliability of our RNA-seq results, we conducted qPCR validation for all the genes mentioned in this study (Supplementary Table S9). While there were variations in the degrees of fold change, the majority of the genes exhibited consistent differential expression in the same direction, reinforcing the validity of our findings.

## Discussion

Despite tremendous advances in modern medicine, there are few available drugs that effectively improve liver function or protect against liver damage. Sericin is a natural product that has remarkable antioxidant, anti-inflammatory and anticancer properties and has been extensively used to treat various diseases^7^. Due to the limited information on the extensive effects of sericin on the transcriptome, RNA-seq was utilized to reveal the DEGs and cellular pathways affected by sericin in HepG2 cells. These data not only confirm the hepatoprotective effect of sericin but also reveal several novel genes in different cellular pathways that are regulated by sericin in HepG2 cells. To evaluate the cytotoxicity of sericin, cells were treated with different concentrations of sericin (0, 0.125, 0.25, 0.5, 1 mg/mL). Sericin was not toxic to HepG2 cells. Due to the nontoxicity of sericin, a low concentration (0.125 mg/mL) and a high concentration (1 mg/mL) were selected for the subsequent transcriptome analysis. The RNA-seq analysis detected 60,600 transcripts in the sericin-treated HepG2 cells. DEG analysis, pathway enrichment analyses (GO, KEGG, Reactome), and network analysis (PPIA) were used to compare gene regulation among the HepG2 cell groups (0.125 mg/mL vs. untreated, 1 mg/mL vs. untreated, and 1 mg/mL vs. 0.125 mg/mL).

In the 0.125 mg/mL sericin group, numerous enriched pathways were detected, such as the coagulation cascade pathway *(A2M, SERPINA5),* metallothionein binding metal pathway and response to metals *(MT2A, MT1G, MT1E)* and HDM demethylate histone pathway *(ARID5B, KDM7A, KDM6B)*; these altered pathways exhibited the potent hepatoprotective activity of sericin. Moreover, *BCL6* was found to be a novel target of low-dose sericin treatment. A2M, alpha-2-macroglobulin, is a proteinase inhibitor that inactivates several proteinases and suppresses fibrinolysis by reducing plasmin and kallikrein. Previous studies have reported that an increase in A2M suppresses the catabolism of matrix proteins that cause liver fibrosis^19^. Gangadharan B et al. demonstrated that thioester cleavage of A2M increases slowly with the development of fibrosis^20^. In the present study, downregulation of A2M might reduce liver fibrosis, suggesting that A2M is a novel biomarker target of sericin. *SERPINA5,* Serine Proteinase Inhibitor Clade A Member 5, is a major anticoagulant protease that can suppress activated protein C. Fan et al. showed that SERPINA5 enhances tumor cell proliferation by regulating the PI3K/AKT/mTOR signaling pathway in gastric cancer^21^. Zhang et al. revealed that SERPINA5 was upregulated in liver metastatic cancer tissues^22^. These findings support our findings that the downregulation of SERPINA5 might be associated with a reduction in liver damage and the alleviation of liver cancer. Interestingly, the expression of genes encoding metallothionein was upregulated upon low-dose sericin administration. Metallothionein is a cysteine-rich protein that plays a vital role in metal homeostasis, heavy metal toxicity, DNA damage and oxidative stress protection. Abnormal expression of metallothionein genes is noted in several cancers and is related to tumor formation, metastasis, drug resistance and poor prognosis. Previous studies have shown that metallothionein 2A *(MT2A)* is downregulated in gastric cancer cells and exerts its anti-gastric cancer effect by binding to MZF1 to target NFKBIA^23^. In hepatoma cells, *MT2A* is also downregulated and plays a cancer suppressive role^24^. In addition, metallothionein 1G *(MT1G)* is reduced in human HCC tissues due to promoter hypermethylation^25^, and metallothionein 1E *(MT1E)* is downregulated in hepatocellular carcinoma (HCC), which induces apoptosis and suppresses the metastasis of HCC^26^. These findings are consistent with our study in that an increase in the expression of metallothionein genes might indicate an anticancer effect of sericin. Histone demethylases are involved in regulating cellular processes such as chromatin structure and transcription^27^. Kim et al. revealed that overexpression of the histone demethylase KDM7A induces hepatic steatosis via upregulation of DGAT2 by erasing H3K9me2 and H3K27me2 on the promoter^28^. Tang et al. reported that the histone demethylase KDM6B promotes epithelial–mesenchymal transition, migration, invasion, and stemness in HCC^29^. He et al. showed that the upregulation of AT-rich interaction domain 5B (*ARID5B)* is associated with the activation of hepatic stellate cells, which leads to liver fibrosis^30^. These results suggested that the downregulation of genes related to histone demethylation might play a role in the hepatoprotective effect of sericin.

Surprisingly, administration of 1 mg/mL sericin promoted pathways involved in lipid metabolism and atherosclerosis, MAPK signaling, selective autophagy, microautophagy, and autophagy. Accordingly, the *POU2F1* and *APOB* genes involved in lipid metabolism were downregulated in sericin-treated cells. POU domain class 2 transcription factor 1 (POU2F1) is a ubiquitous transcription factor that regulates the transcription of target genes related to the cell cycle^31^. Zhong et al. reported that *POU2F1* overexpression is positively related to aggressive phenotypes and poor survival in patients with HCC. *POU2F1* is modulated by the AKT pathway and promotes aggressive HCC phenotypes by regulating the transcription of epithelial-to-mesenchymal transition genes^32^. Zhu et al. reported that *POU2F1* enhances the growth and metastasis of HCC via the FAT1 signaling pathway^33^. These previous findings support our findings that downregulation of POU2F2 might alleviate HCC. Apolipoprotein B (APOB) plays a critical role in human lipoprotein metabolism. Gudowska et al. reported that an increase in *APOB* is associated with viral hepatitis and alcoholic cirrhosis^34^. Crooke et al. demonstrated that suppression of APOB by antisense oligonucleotides reduced LDL cholesterol without causing hepatic steatosis^35^. This result aligns with our finding that a lower APOB might decrease the release of triglyceride-rich lipoprotein, thereby ameliorating hepatic steatosis. In addition, the gene pathway annotation network revealed that *TRAF6, HSPA8, and FGFR1*, which are involved in MAPK signaling, were downregulated. TNF receptor associated factor 6 (TRAF6) is a key mediator of NF-κB signaling and is related to the IL-1R/TLR pathway and restrains the phosphorylation of IκBα^36^. Once activated, TRAF6 initiates an inflammatory cascade amplification, leading to tissue damage and organ dysfunction^37^. Therefore, the downregulation of TRAF6 suggested that sericin could reduce the inflammatory cascade and protect the liver from damage. Heat shock protein 70 (HSP70) and its major cochaperones, including cytosolic HSPA8, are related to various phenotypes of tumorigenesis, including proliferation, invasion, and metastasis^38^. Consistent with our study, downregulating *HSPA8* upon sericin treatment possibly helps to prevent liver cancer. Fibroblast growth factor receptors (FGFRs) are master mediators of a broad spectrum of cellular and developmental processes involving apoptosis, proliferation, and angiogenesis. FGFR1 has been reported to promote both hepatic stellate cell (HSC) activation and proliferation^39^. This report correlates with our findings that *FGFR1* downregulation upon sericin treatment might suppress HSC activation. Notably, sericin regulates autophagy-related genes *(MAP1LC3B, DYNC1H1* and *HSPA5)*. Autophagy is a cellular process that removes polyubiquitinated protein aggregates and mitochondria^40^. Microtubule-associated protein 1 light chain 3 (MAP1LC3B) is a major marker of autophagy that acts as an autophagy receptor for autophagosome elongation in mammalian cells^41^. Yu et al. demonstrated that low expression of MAP1LC3B predicts lymph node metastasis and poor prognosis in gastric cancer patients^42^. Dynein cytoplasmic 1 heavy chain 1 (DYNC1H1) is a protein-coding gene encoding the cytoplasmic dynein heavy chain family. Wang et al. reported that DYNC1H1 expression influences liver hepatocellular carcinoma initiation and progression and the immune microenvironment^43^. Yang et al. indicated that high levels of HSPA5 are associated with earlier recurrence of HCC^44^. Notably, high-dose sericin administration attenuated the expression of liver-associated disease genes, which was accompanied by, at least, upregulation of *MAP1LC3B* and downregulation of *DYNC1H1* and *HSPA5*, which are autophagy-related genes.

Notably, one gene that regulates lipid metabolism and atherosclerosis pathways was affected by sericin at 0.125 mg/mL to 1 mg/mL. The *O*-linked β-*N*-acetylglucosamine transferase (OGT) is responsible for the addition of GlcNAc moieties to select serine and threonine residues on nuclear and cytosolic proteins and is involved in a number of cellular functions. Alterations in OGT have been found to be related to many pathologies, such as diabetes, cardiovascular disease, neurodegeneration and cancer^45^. Lynch et al. showed that OGT levels are usually increased in cancer. A decrease in OGT in cancer cells leads to growth reduction and metastasis^46^. This study supported our findings that the downregulation of *OGT* upon sericin administration at both high and low doses might reduce liver-associated diseases.

The TFs obtained from the DEGs were identified to further study the molecular regulatory network activated or deactivated by sericin administration. Alterations in TF activity were calculated using DEG data. Based on the TF activity prediction, numerous transcription factor genes and their downstream targets were shown to be associated with cellular differentiation, cell proliferation, the inflammatory response, oxidative stress and lipid metabolism and were affected by both low and high doses of sericin. This finding suggested the modulation of crucial biological functions, potentially alleviating liver injury. CEBPA, STAT1, TFAP2C and GATA3 TFs were predicted to be deactivated, while the NFE2L2 TF was predicted to be activated upon low-dose sericin administration. CEBPA, a TF belonging to the CCAAT/enhancer-binding protein family, plays a vital role in hepatic glucose and lipid homeostasis as well as in the maintenance of cell proliferation^47,48^. Interestingly, the NFE2L2 TF was also predicted to be activated upon high-dose sericin administration. Similarly, deactivation of the STAT1*,* TFAP2C and GATA3 TFs was observed upon sericin administration. In contrast, only the CREB1 TF exhibited a difference in expression pattern following low-dose sericin administration. Previous studies have shown that CEBPA-null mice exhibit glucose intolerance, decreased serum cholesterol levels, and hepatic steatosis^47^. Strikingly, *CEBPA* has been reported to be upregulated in a subset of HCCs and to have growth-promoting effects on HCC cells^49^. These opposing roles of CEBPA in HCC cell subsets may be determined by epigenetic regulatory mechanisms^50^. This trend is consistent with our study, which indicated that cell growth decreased due to reduced CEBPA expression. STAT1 is a central signaling constituent of IFN and plays vital roles in antitumor activity, antiviral defense, induction of liver injury and inflammation and inhibition of liver regeneration^51^. Accumulated research data suggest that suppression of hepatic STAT1 activation through genetic modification of various genes prevents concanavalin A-induced liver injury^52,53^. These findings support our findings that deactivation of STAT1 upon sericin administration may ameliorate liver inflammation and liver injury. TFAP2C, a member of the AP2 transcription factor family, is critical for maintaining cellular homeostasis; regulating proliferation and apoptosis; and promoting embryonic development^54^. A previous study revealed that transgenic overexpression of *Tcfap2c* in the liver promoted proliferation and apoptosis and induced hepatic steatosis, leading to liver failure^55^. Hence, we speculated that the deactivation of the TFAP2C TF upon sericin administration might suppress proliferation, reduce hepatic steatosis and subsequently alleviate liver damage. GATA3, a member of the GATA family, plays a crucial role in inflammatory and humoral immune responses^56^. Its expression was previously shown to be associated with impaired adipogenesis, and it contributes to the induction of inflammatory cytokine production in adipose tissue^57^. The inhibition of GATA3 expression improved adipocyte differentiation, reduced inflammation and reversed insulin resistance^58^. In addition, suppression of GATA3 increased the mRNA and protein levels of lipase A, which serves as a biomarker of liver fibrosis^59^. Our study indicated that the GATA3 TF is likely deactivated upon sericin administration, consistent with the findings of previous research. Therefore, we inferred that sericin might exert its anti-liver fibrosis effect by targeting GATA3 and reducing inflammatory molecules in the liver. NFE2L2 plays a role in activating the oxidative stress defense mechanism by promoting antioxidant and detoxifying enzymes to prevent cells from diverse oxidative insults^60^. Many studies have reported that the hepatoprotection mechanism against oxidative stress-induced HCC primarily involves the modulation of various genes, including those involved in glutathione biosynthesis, which prevents oxidative damage^61,62^. These findings support our study, suggesting that sericin significantly activates NFE2L2 TF in the HepG2 cell line, indicating that its hepatoprotective effects can be attributed to its antioxidant effects. CREB1 is involved in gene regulation in response to various extracellular signals^63^, and its transcriptional activity is modulated by Ser133 phosphorylation^64^. Previous studies have implicated CREB1 in fibrogenesis. It facilitates high glucose-promoted renal tubulointerstitial fibrosis in diabetic nephropathy^65^. Additionally, activation of CREB1 is markedly increased in CCl_4_-induced liver fibrosis^66^, and in alcoholic liver fibrosis^67^. Thus, our finding of predicted deactivation of the CREB1 TF upon sericin treatment aligns with the findings of previous studies suggesting that downregulation of CREB1 may contribute to the recovery of hepatic fibrosis. To this end, these analyses showed that sericin could alleviate liver injury by modulating transcription factor activity.

This study further revealed that sericin significantly mitigated APAP-induced cell death in HepG2 cells, a known effect of APAP, as documented in previous studies^68^. Sericin also modulates the expression of genes linked to lipid metabolism *(POU2F1, APOB, OGT),* MAPK signaling *(TRAF6)*, and autophagy *(HSPA5).* Notably, the ability of sericin to counteract APAP-induced damage was evident in its regulation of key genes, with a pronounced effect on *TRAF6* expression, which is typically upregulated in response to liver injury^69^. Taken together, our findings on sericin are the first to show that sericin has many hepatoprotective effects on hepatocytes, promoting liver function and alleviating liver-associated diseases.

## Conclusions

A hepatic transcriptome analysis showed that sericin administration leads to significant transcriptional changes in human hepatocellular carcinoma cells. Computational analysis revealed the effects of sericin at various doses: a lower dose influenced the complement and coagulation cascades, metallothionine metal binding, and histone demethylase activity, whereas a higher dose affected lipid metabolism, atherosclerosis, MAPK signaling and autophagy. Gene network analysis revealed novel targets, highlighting transcription factors involved in the effects of sericin and confirming its protective effects. Collectively, these findings demonstrate the potent hepatoprotective effects of sericin via multiple pathways, offering valuable insights for therapeutic drug development.

## Materials and methods

### Sericin extraction

Silkworm cocoons (*Bombyx mori*) were obtained from Chul Thai Silk Co., Ltd., Phetchabun Province, Thailand. Sericin was extracted from fresh cocoon shells by autoclaving in purified water at 120 °C for 60 min. The supernatant was collected, filtered, frozen, and then lyophilized.

### Cell culture and viability assessment

HepG2 cells (ATCC, HB8065, USA) were maintained in DMEM supplemented with 10% fetal bovine serum, 100 units/mL penicillin, and 100 µg/mL streptomycin at 37 °C and 5% CO_2_ in a humidified environment. For detachment, the cells were incubated with trypsin. A sericin stock solution was prepared in distilled water, while acetaminophen (APAP) (Tylenol, Johnson & Johnson, Thailand) was dissolved in absolute ethanol. These stock solutions were subsequently added to the culture medium to reach the desired concentrations. The effect of sericin on HepG2 cell viability was evaluated using the MTT assay. Cells were seeded in 96-well plates and allowed to incubate for 24 h. Subsequently, the cells were treated with various concentrations of sericin (0, 0.125, 0.25, 0.5, or 1 mg/mL) and APAP (0, 2.5, 5, 10, or 20 mM) for an additional 24 h. After treatment, the medium was discarded, and each well was incubated with MTT solution (5 mg/mL) for 4 h. The formazan crystals that formed were dissolved in 100 µL of DMSO per well. The absorbance was subsequently measured at 570 nm using a spectrophotometer.

### RNA isolation

Total RNA was isolated from the HepG2 cells using the RNeasy Mini Kit (Qiagen GMBH, Hilden, Germany) according to the manufacturer’s instructions, yielding approximately 20 μg of total RNA. The amount of total RNA was determined using a NanoDrop™ 8000 spectrophotometer (Thermo Fisher Scientific, USA). The quality of the RNA preparation was evaluated using an Agilent RNA 6000 Nano Kit on an Agilent Bioanalyzer 2100 system (Agilent, Palo Alto, CA). RNA samples with an integrity number (RIN) greater than 9.0 (and a 28S/18S ratio ranging from 1.8 to 2.2) were used for library construction.

### mRNA library preparation and sequencing

Total RNA (1 μg) was used for subsequent library preparation. Poly(A) mRNA isolation was performed using oligo (dT) beads. mRNA fragmentation was carried out using divalent cations and at high temperature. Priming was performed using random primers. First-strand cDNA and second-strand cDNA were synthesized. The purified double-stranded cDNA was then treated to repair both ends, after which dA was added to one reaction, followed by T-A ligation to add adaptors to both ends. Selection of the size of the adaptor-ligated DNA was then performed using DNA clean beads. PCR was subsequently performed for each sample using the P5 and P7 primers, after which the PCR products were validated. Libraries with different indices were multiplexed and loaded on an Illumina HiSeq/Illumina NovaSeq/MGI2000 instrument for sequencing using a 2 × 150 paired-end (PE) configuration according to the manufacturer’s instructions, with three technical replicates.

### Preprocessing of RNA-Seq data

Low-quality reads were filtered out according to CutAdapt software (V1.9.1). The filtering process was used to produce high-quality cleaned reads by removing low-quality reads, sequences containing an unknown base N, adapter sequences, reads with more than 10% of skipped bases, and reads with an average quality score less than 20. The remaining reads were mapped onto the human reference genome (UCSC, NCBI, ENSEMBL) using the aligner software HISAT2 (v2.0.1). The transcripts in FASTA format were subsequently converted from a known gff annotation file and indexed to a reference gene file. The gene expression levels were measured using HTSeq (v0.6.1).

### Differentially expressed genes (DEGs) analysis

Differential expression analysis was performed using the DESeq2 Bioconductor package, which is based on a model utilizing a negative binomial distribution. This approach incorporates data-driven prior distributions for estimates of dispersion and logarithmic fold changes. The adjusted p-value (padj) of genes, corrected with the Benjamini–Hochberg method for multiple comparison adjustment, was set at < 0.05 to identify DEGs.

### Functional annotation and classification

Differentially expressed genes with an FDR less than 0.05 and a log2-fold change greater than 1.25 or less than − 1.25 were inputted into PathfindR. The KEGG pathway^70–72^, Reactome and GO databases were used for pathway analysis and functional classification. Only pathways and functional groups with p-values less than 0.05 were discussed. The active subnetworks were identified in a Biogrid protein‒protein interaction network and then subjected to pathway enrichment analysis in accordance with the database.

### Transcription factor analysis

The sericin treatment group included a list of DEGs enriched with transcription factor (TF) enrichment, which was determined via decoupleR. The TF regulatory network was analyzed with the meta-database OmniPath via DecoupleR. These transcription factor activities were studied based on DoRothEA, which is a comprehensive prior knowledge resource obtaining curated TFs and their downstream targets. TF activity scores were calculated from z-transformed expression values (FDR less than 0.05).

### Validation of hepatic gene expression following sericin treatment

RT‒qPCR was used to validate the top 10 differentially expressed and novel genes from RNA–seq and to measure gene expression in hepatoprotective experiments. RNA was reverse transcribed using a RevertAid First Strand cDNA Synthesis Kit (Thermo Fisher Scientific, Waltham, MA, USA) according to the manufacturer’s instructions. RT‒qPCR was performed using Luna^®^ Universal qPCR Master Mix (Biolabs, USA) according to the manufacturer’s instructions. All the specific primers used were designed using Oligo 7 primer analysis software (Supplementary Table S10), and the thermal cycling conditions are provided in Supplementary Table S11. The Ct value was normalized to that of the housekeeping gene GAPDH. Relative gene expression was calculated using the 2^−ΔΔCt^ method. In addition, melting curves were generated to confirm the specificity and consistency of the products.

### Supplementary Information

Supplementary Figure S1.

Supplementary Figure S2.

Supplementary Figure S3.

Supplementary Figure S4.

Supplementary Table S1.

Supplementary Table S2.

Supplementary Table S3.

Supplementary Table S4.

Supplementary Table S5.

Supplementary Table S6.

Supplementary Table S7.

Supplementary Table S8.

Supplementary Table S9.

Supplementary Table S10.

Supplementary Table S11.

## Data Availability

The datasets generated and analyzed during the study are available in the Gene Expression Omnibus (GEO) repository, accession number GSE229079.

## Abbreviations

A2M: Alpha-2-macroglobulin
APAP: Acetaminophen
APOB: Apolipoprotein B
ARID5B: AT-rich interaction domain 5B
CAT: Catalase
CREB1: CAMP responsive element binding protein 1
DEGs: Differentially expressed genes
FGFR1: Fibroblast growth factor receptors
GATA3: GATA binding protein 3
GO: Gene Ontology
GSH-PX: Plasma glutathione peroxidase enzymes
HCC: Hepatocellular carcinoma
HDMs: Histone demethylate
HSPA8: Heat shock protein family A (Hsp70) member 8
IR: Insulin resistance
IRS-1: Insulin receptor substrate-1
KEGG: Kyoto encyclopedia of genes and genomes
MAPK: Mitogen-activated protein kinase
MT1E: Metallothionein 1E
MT1G: Metallothionein 1G
MT2A: Metallothionein 2A
NFE2L2: NFE2 like bZIP transcription factor 2
OGT: O-linked β-N-acetylglucosamine transferase
PI3K: Phosphatidylinositol-3-kinase
POU2F1: POU domain class 2 transcription factor 1
PPIN: Protein–protein interaction network
SERPINA5: Serine proteinase inhibitor clade A member 5
SOD: Superoxide dismutase
STAT1: Signal transducer and activator of transcription 1
TFAP2C: Transcription factor AP-2 gamma
TRAF6: TNF receptor associated factor 6
