# Supplementary material for: Transcriptomic screening of novel targets of sericin in human hepatocellular carcinoma cells
Source: Sci Rep. 2024 Mar 5;14:5455. doi: 10.1038/s41598-024-56179-y (PMC10914811; doi:10.1038/s41598-024-56179-y)
Supplement: Supplementary file 1 — Supplementary Figure S1. [file 41598_2024_56179_MOESM1_ESM.pdf]

**Supplementary Figure S1**

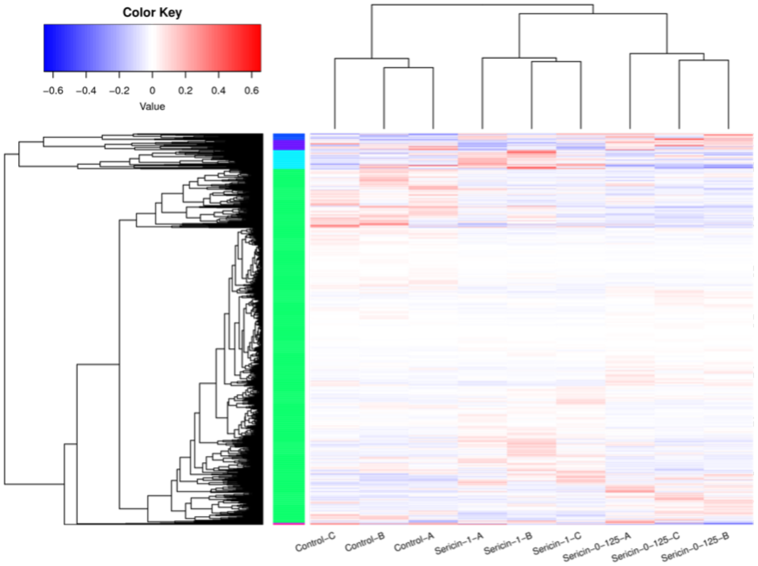


Hierarchical clustering of normalized gene expression for sericin treatment
