# Supplementary material for: Transcriptomic screening of novel targets of sericin in human hepatocellular carcinoma cells
Source: Sci Rep. 2024 Mar 5;14:5455. doi: 10.1038/s41598-024-56179-y (PMC10914811; doi:10.1038/s41598-024-56179-y)
Supplement: Supplementary file 2 — Supplementary Figure S2. [file 41598_2024_56179_MOESM2_ESM.pdf]

Activity of transcription factors inferred from their regulon expression changes upon 0.125 mg/ mL sericin administration

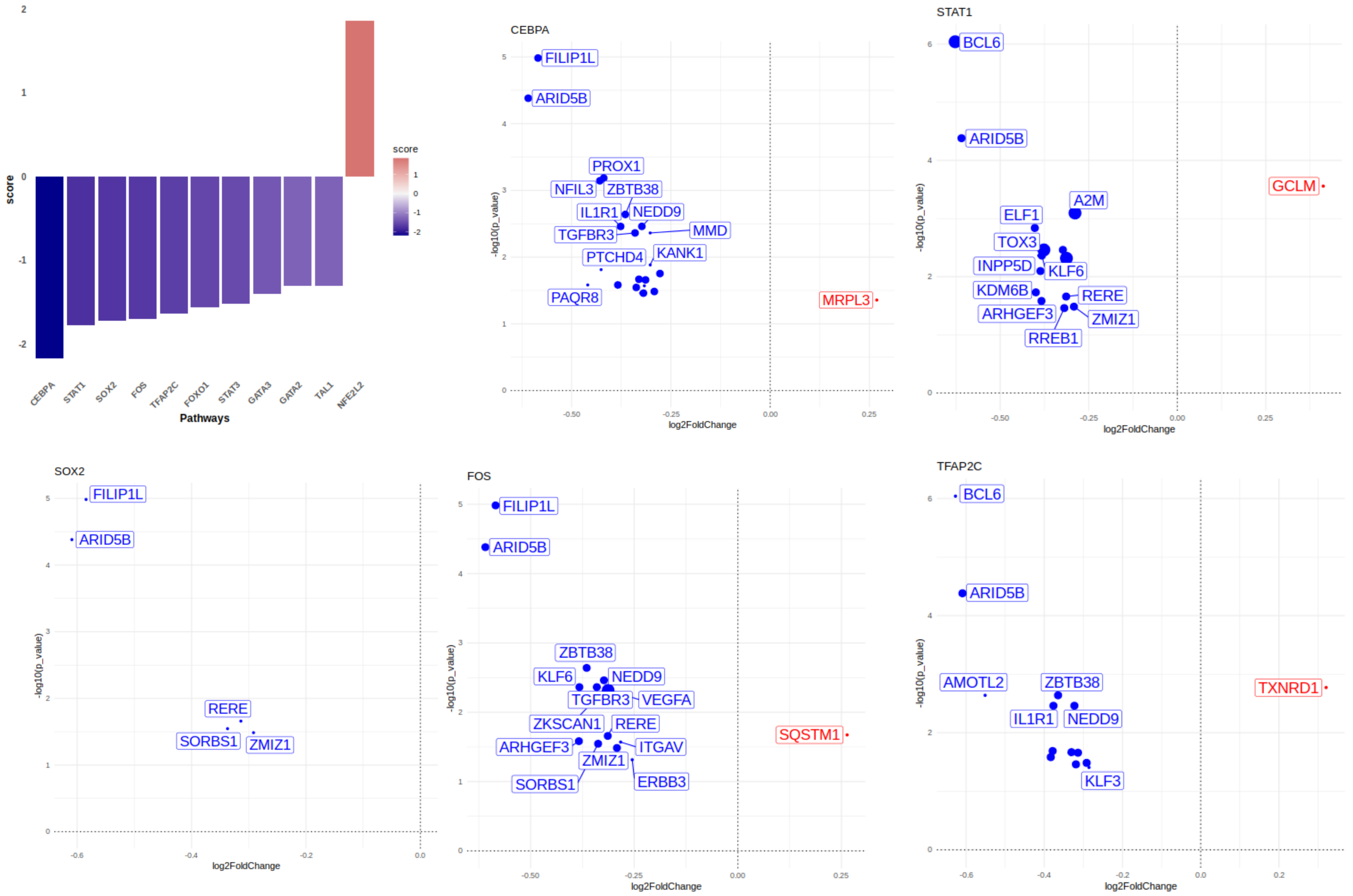

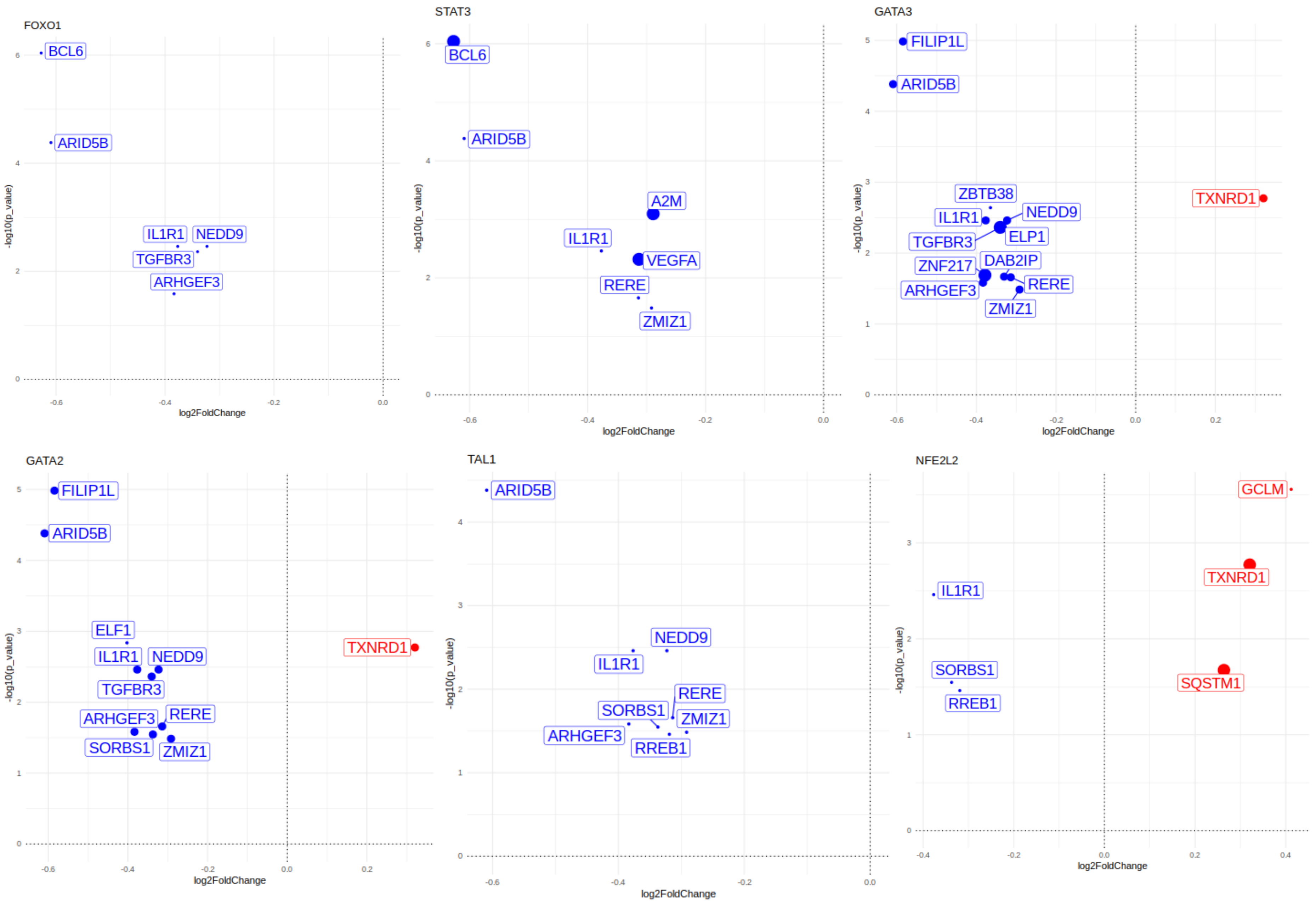
FOXO1
BCL6
ARID5B
IL1R1
NEDD9
TGFBR3
ARHGEF3
-log10(p_value)
log2FoldChange
STAT3
BCL6
ARID5B
A2M
IL1R1
VEGFA
RERE
ZMIZ1
GATA3
FILIP1L
ARID5B
ZBTB38
NEDD9
IL1R1
TGFBR3
ELP1
ZNF217
DAB2IP
ARHGEF3
RERE
ZMIZ1
TXNRD1
GATA2
FILIP1L
ARID5B
ELF1
IL1R1
NEDD9
TGFBR3
ARHGEF3
RERE
SORBS1
ZMIZ1
TXNRD1
TAL1
ARID5B
NEDD9
IL1R1
RERE
SORBS1
ZMIZ1
ARHGEF3
RREB1
NFE2L2
GCLM
TXNRD1
IL1R1
SORBS1
RREB1
SQSTM1
