# Supplementary material for: Transcriptomic screening of novel targets of sericin in human hepatocellular carcinoma cells
Source: Sci Rep. 2024 Mar 5;14:5455. doi: 10.1038/s41598-024-56179-y (PMC10914811; doi:10.1038/s41598-024-56179-y)
Supplement: Supplementary file 3 — Supplementary Figure S3. [file 41598_2024_56179_MOESM3_ESM.pdf]

Activity of transcription factors inferred from their regulon expression changes upon 1 mg/ mL sericin administration

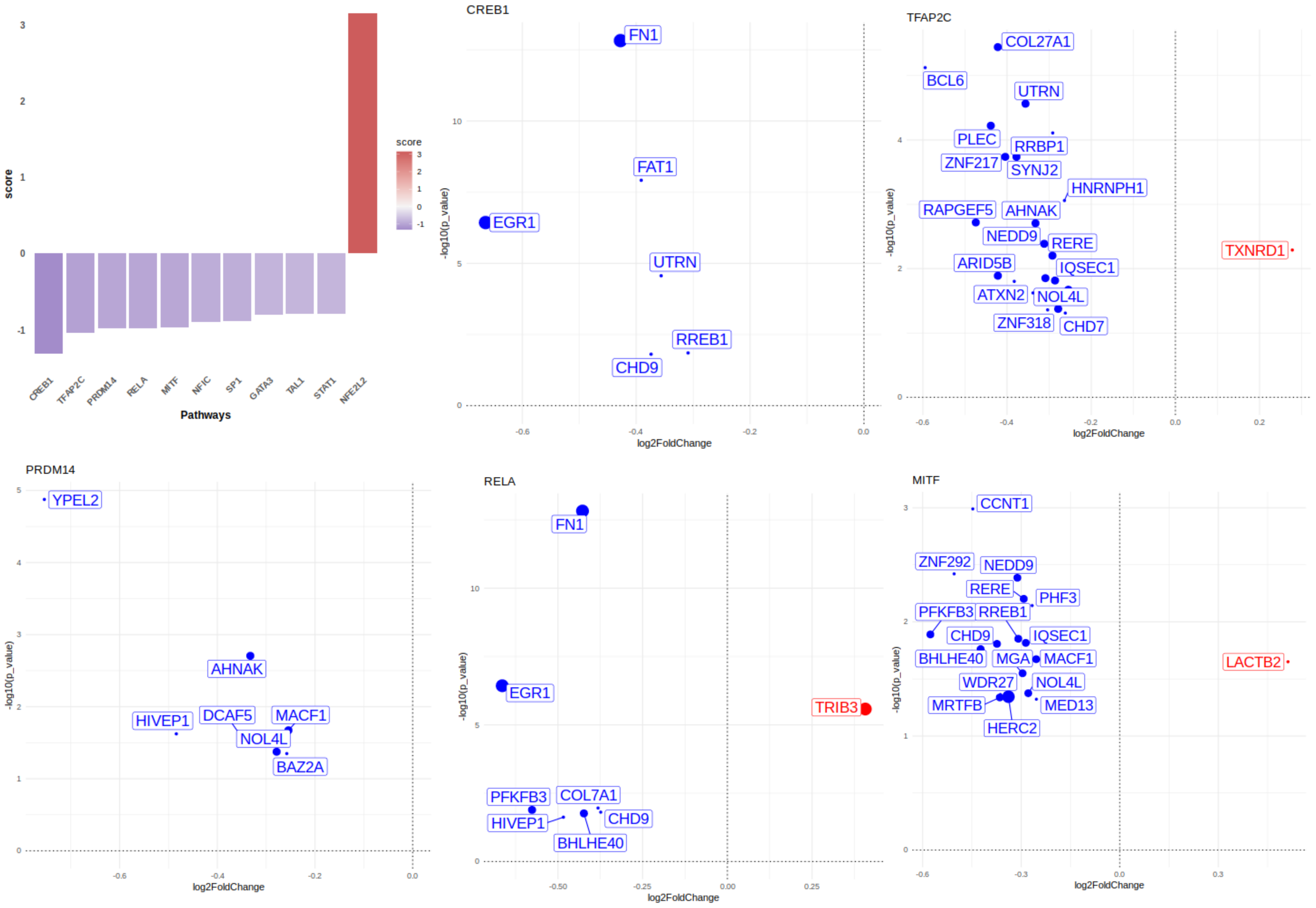

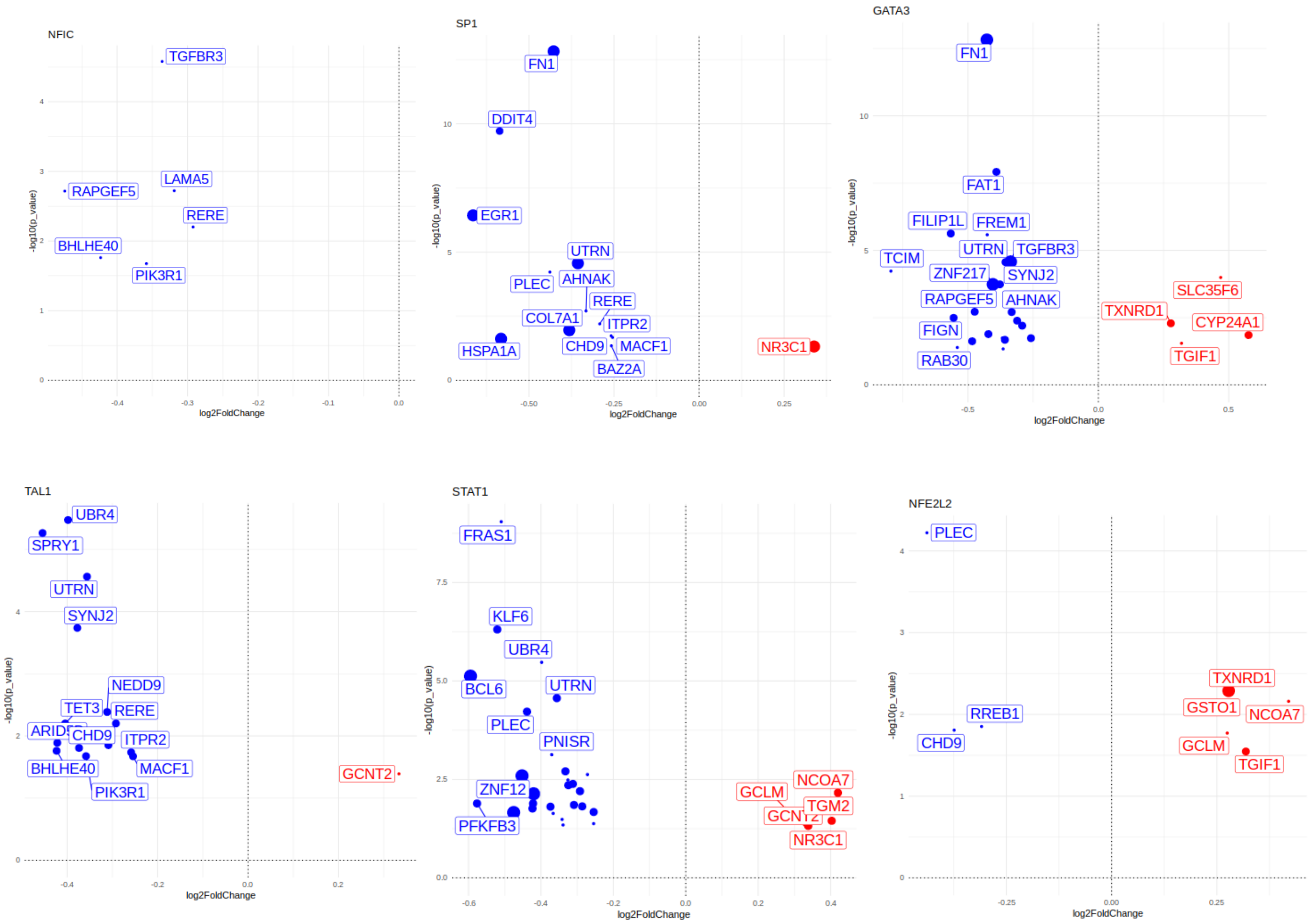
NFIC
TGFBR3
LAMA5
RAPGEF5
RERE
BHLHE40
PIK3R1
-log10(p_value)
log2FoldChange
SP1
FN1
DDIT4
EGR1
UTRN
PLEC
AHNAK
RERE
COL7A1
ITPR2
HSPA1A
CHD9
MACF1
BAZ2A
NR3C1
-log10(p_value)
log2FoldChange
GATA3
FN1
FAT1
FILIP1L
FREM1
TCIM
UTRN
TGFBR3
ZNF217
SYNJ2
RAPGEF5
AHNAK
SLC35F6
TXNRD1
CYP24A1
FIGN
TGIF1
RAB30
-log10(p_value)
log2FoldChange
TAL1
UBR4
SPRY1
UTRN
SYNJ2
NEDD9
TET3
RERE
CHD9
ITPR2
BHLHE40
MACF1
GCNT2
PIK3R1
-log10(p_value)
log2FoldChange
STAT1
FRAS1
KLF6
UBR4
BCL6
UTRN
PLEC
PNISR
ZNF12
NCOA7
GCLM
TGM2
PFKFB3
NR3C1
-log10(p_value)
log2FoldChange
NFE2L2
PLEC
TXNRD1
RREB1
GSTO1
NCOA7
CHD9
GCLM
TGIF1
-log10(p_value)
log2FoldChange
