# Supplementary figures and images for: Transcriptomic screening of novel targets of sericin in human hepatocellular carcinoma cells
Source: Sci Rep. 2024 Mar 5;14:5455. doi: 10.1038/s41598-024-56179-y (PMC10914811; doi:10.1038/s41598-024-56179-y)

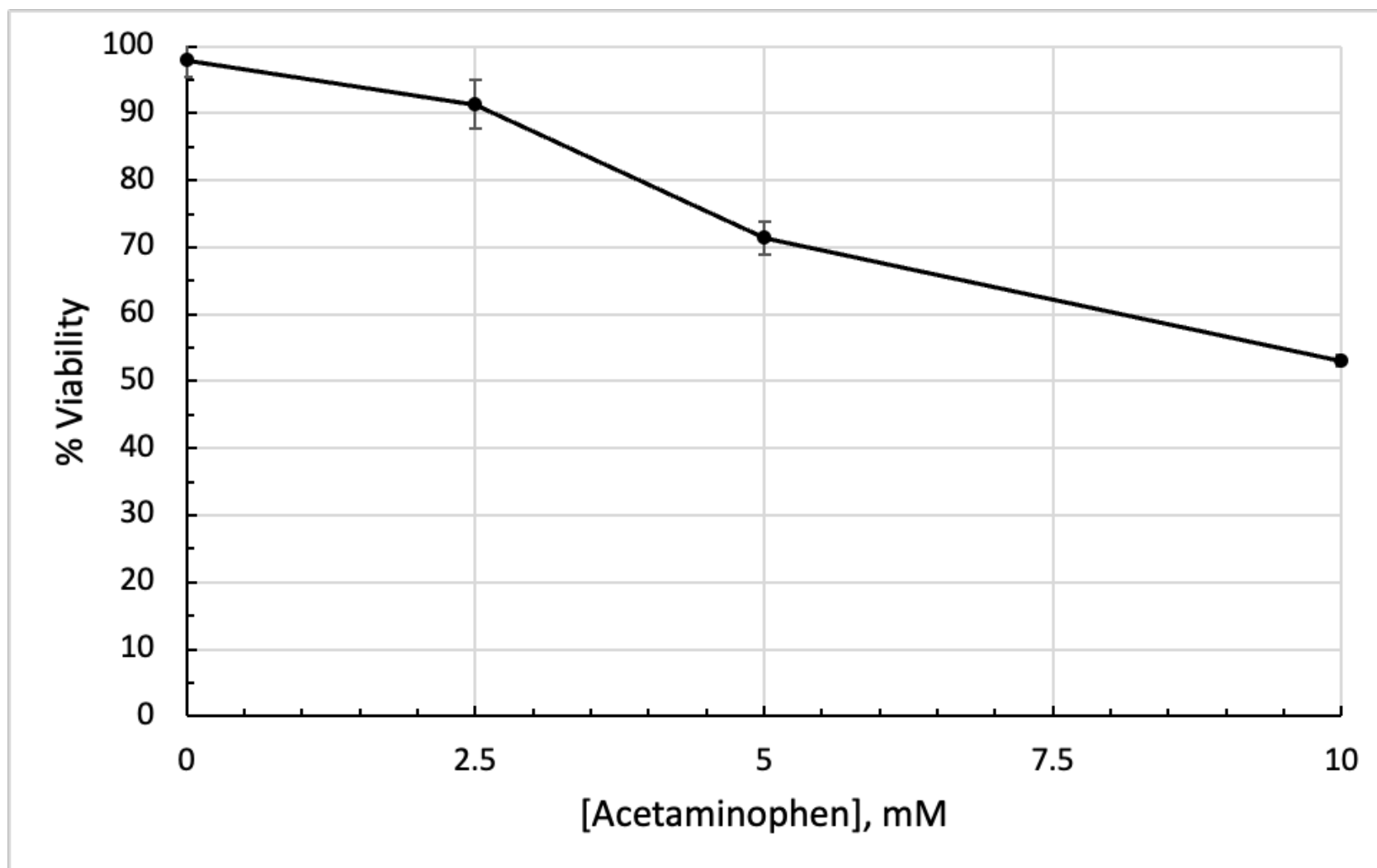

Figure S4: Cytotoxic effect of Acetaminophen

Supplement: Supplementary file 4 — Supplementary Figure S4. [file 41598_2024_56179_MOESM4_ESM.pdf]
