# Supplementary material for: Transcriptomic screening of novel targets of sericin in human hepatocellular carcinoma cells
Source: Sci Rep. 2024 Mar 5;14:5455. doi: 10.1038/s41598-024-56179-y (PMC10914811; doi:10.1038/s41598-024-56179-y)
Supplement: Supplementary file 6 — Supplementary Table S2. [file 41598_2024_56179_MOESM6_ESM.pdf]

GO-Analysis (0.125 mg/ mL vs untreated)

ID	Term_Description	Fold_Enrichment	occurrence	support	lowest_p	highest_p	Up_regulated	Down_regulated
GO:2000556 (GO:2000556)	positive regulation of T-helper 1 cell cytokine production	53.041463	5	0.0050761	0.00063	0.00063		IL1R1
GO:0061419 (GO:0061419)	positive regulation of transcription from RNA polymerase II promoter in response to hypoxia	53.041463	8	0.0059854	0.00127	0.00127		VEGFA
GO:0014912 (GO:0014912)	negative regulation of smooth muscle cell migration	33.150915	4	0.0050761	0.00178	0.00178	SERPINE1	
GO:0006111 (GO:0006111)	regulation of gluconeogenesis	37.886760	1	0.0107527	0.00266	0.00266		OGT
GO:0000423 (GO:0000423)	mitophagy	44.201220	7	0.0054348	0.00317	0.00317	SQSTM1	
GO:0038132 (GO:0038132)	neuregulin binding	106.082927	9	0.0163043	0.00317	0.00317		ERBB3, ITGAV
GO:0043125 (GO:0043125)	ErbB-3 class receptor binding	53.041463	9	0.0065359	0.00317	0.00317		ERBB3
GO:0006335 (GO:0006335)	DNA replication-dependent nucleosome assembly	33.150915	1	0.0107527	0.00355	0.00355	ASF1A	
GO:0016600 (GO:0016600)	flotillin complex	37.886760	8	0.0065359	0.00444	0.00444		SORBS1
GO:0097192 (GO:0097192)	extrinsic apoptotic signaling pathway in absence of ligand	88.402439	9	0.0163043	0.00476	0.00476		ERBB3, ITGAV
GO:0030705 (GO:0030705)	cytoskeleton-dependent intracellular transport	44.201220	5	0.0054348	0.00476	0.00476	TUBA1B	
GO:0000978 (GO:0000978)	RNA polymerase II cis-regulatory region sequence-specific DNA binding	7.444416	10	0.0119707	0.00490	0.00490		CHD2, KLF6, ELF1, NFIL3, PROX1, RREB1, NR2F1, ZNF217
GO:0003700	DNA-binding	5.375824	10	0.0065359	0.00570	0.00570		BCL6, ELF1,

(GO:0003700)	transcription factor activity							NFIL3, NR2F1, ZNF217, ZBTB38
GO:0006336 (GO:0006336)	DNA replication-independent nucleosome assembly	26.520732	4	0.0050761	0.00571	0.00571	ASF1A	
GO:0042730 (GO:0042730)	fibrinolysis	24.109756	8	0.0059854	0.00697	0.00697	SERPINE1	
GO:0043065 (GO:0043065)	positive regulation of apoptotic process	3.900108	4	0.0066926	0.00755	0.04732	SQSTM1	BCL6, DAB2IP
GO:0045892 (GO:0045892)	negative regulation of transcription, DNA-templated	4.606579	6	0.0068260	0.00846	0.01946		BCL6, NFIL3, PROX1, ZNF217, ARID5B, DAB2IP, ZBTB38
GO:0070498 (GO:0070498)	interleukin-1-mediated signaling pathway	15.600430	5	0.0101523	0.00862	0.00862		IL1R1
GO:0007017 (GO:0007017)	microtubule-based process	33.150915	5	0.0054348	0.00887	0.00887	TUBA1B	
GO:0045926 (GO:0045926)	negative regulation of growth	61.201689	10	0.0130719	0.00989	0.01648	MT1E, MT1G, MT2A	
GO:0043235 (GO:0043235)	receptor complex	6.120169	8	0.0059854	0.01035	0.01035		ERBB3, LIFR, TGFBR3
GO:0071294 (GO:0071294)	cellular response to zinc ion	53.041463	10	0.0098808	0.01331	0.02218	MT1E, MT1G, MT2A	
GO:0006974 (GO:0006974)	cellular response to DNA damage stimulus	4.101144	10	0.0065359	0.01389	0.01389		BCL6, ZBTB40, ZBTB38
GO:0006334 (GO:0006334)	nucleosome assembly	16.575457	3	0.0050761	0.01521	0.01521	ASF1A	
GO:0070555 (GO:0070555)	response to interleukin-1	11.050305	5	0.0101523	0.01750	0.01750		IL1R1
GO:0015026 (GO:0015026)	coreceptor activity	29.467480	1	0.0050761	0.01939	0.01939		ITGAV, TGFBR3
GO:0043548 (GO:0043548)	phosphatidylinositol 3-kinase binding	26.520732	1	0.0050761	0.01996	0.01996		DAB2IP
GO:0004867 (GO:0004867)	serine-type endopeptidase inhibitor activity	30.600844	8	0.0110389	0.02060	0.04117	SERPINE1	A2M, SERPINA5
GO:0034144 (GO:0034144)	negative regulation of toll-like receptor 4 signaling pathway	33.150915	10	0.0065359	0.02129	0.02660		DAB2IP
GO:0045862 (GO:0045862)	positive regulation of proteolysis	13.958280	4	0.0101523	0.02167	0.02167		OGT
GO:0043149 (GO:0043149)	stress fiber assembly	17.680488	7	0.0054348	0.02218	0.02218		SORBS1

GO:0080182 (GO:0080182)	histone H3-K4 trimethylation	20.400563	9	0.0065359	0.02471	0.02471		OGT
GO:0035331 (GO:0035331)	negative regulation of hippo signaling	40.801126	9	0.0065359	0.02471	0.02471		DLG5, AJUBA
GO:0050431 (GO:0050431)	transforming growth factor beta binding	25.257840	1	0.0050761	0.02661	0.02661		ITGAV, TGFBR3
GO:0031625 (GO:0031625)	ubiquitin protein ligase binding	3.301336	1	0.0050761	0.02666	0.02666	SQSTM1, TUBA1B	ERBB3
GO:0032435 (GO:0032435)	negative regulation of proteasomal ubiquitin-dependent protein catabolic process	11.530753	1	0.0107527	0.03206	0.03206		OGT
GO:0017134 (GO:0017134)	fibroblast growth factor binding	29.467480	8	0.0059854	0.03231	0.03231		ITGAV, KLB
GO:0019838 (GO:0019838)	growth factor binding	44.201220	9	0.0203046	0.03231	0.04843		A2M, ERBB3, LIFR
GO:0071901 (GO:0071901)	negative regulation of protein serine/threonine kinase activity	17.680488	9	0.0065359	0.03325	0.03325		DAB2IP
GO:0046627 (GO:0046627)	negative regulation of insulin receptor signaling pathway	11.050305	3	0.0050761	0.03497	0.03497		KANK1
GO:0051219 (GO:0051219)	phosphoprotein binding	9.822493	1	0.0107527	0.04446	0.04446		TOX3
GO:0051301 (GO:0051301)	cell division	14.733740	5	0.0054348	0.04843	0.04843	TUBA1B	
