# Supplementary material for: Transcriptomic screening of novel targets of sericin in human hepatocellular carcinoma cells
Source: Sci Rep. 2024 Mar 5;14:5455. doi: 10.1038/s41598-024-56179-y (PMC10914811; doi:10.1038/s41598-024-56179-y)
Supplement: Supplementary file 7 — Supplementary Table S3. [file 41598_2024_56179_MOESM7_ESM.pdf]

GO-Analysis (1 mg/ mL vs untreated)

ID	Term_Description	Fold_Enrichment	occurrence	support	lowest_p	highest_p	Up_regulated	Down_regulated
GO:0016607 (GO:0016607)	nuclear speck	4.691201	10	0.1045265	1.4e-12	3.2e-07	NR3C1	HSPA1A, PNN, RREB1, SRSF5, ZNF217, DDX39B, AKAP17A, PRPF4B, RBM39, SRRM1, TCERG1, BAZ2A, FNBP4, SRRM2, PNISR, LUC7L3, RBM25, SAP130, SREK1
GO:0045892 (GO:0045892)	negative regulation of transcription, DNA-templated	3.515490	10	0.0295899	2.1e-07	4.5e-03	NR3C1, ID2, KLF10, TRIB3	BCL6, DDIT3, HSPA8, POU2F1, PROX1, TBX3, TRAF6, ZNF12, ZNF217, BHLHE40, BTAF1, ZNF318, RBAK, CREBZF, EPC1, ARID5B
GO:0000978 (GO:0000978)	RNA polymerase II cis-regulatory region sequence-specific DNA binding	3.479719	10	0.0062510	9.8e-06	5.0e-03	NR3C1, TGIF1, KLF10	CHD2, KLF6, DDIT3, ELF1, POU2F1, PROX1, RREB1, TBX3, ZNF217, ONECUT2, NFAT5
GO:0001228 (GO:0001228)	DNA-binding transcription activator activity, RNA polymerase II-specific	3.320491	3	0.0066225	2.0e-05	1.9e-02	NR3C1, KLF10	KLF6, DDIT3, EGR1, ELF1, ONECUT1, RLF, RREB1, TBX3, ONECUT2, NFAT5
GO:0005041 (GO:0005041)	low-density lipoprotein particle receptor activity	19.319218	3	0.0063291	2.7e-05	4.0e-05		LRP1, LRP6, SORL1
GO:0008380 (GO:0008380)	RNA splicing	5.712672	10	0.0187529	3.3e-05	2.1e-03		DDX39B, PRPF4B, SRRM1, TCERG1, LUC7L3
GO:0003700 (GO:0003700)	DNA-binding transcription factor activity	2.153832	3	0.0066225	4.8e-05	3.3e-02	NR3C1, TGIF1, KLF10	BCL6, DDIT3, EGR1, ELF1, ZNF217, NFAT5
GO:0032922 (GO:0032922)	circadian regulation of gene expression	7.327979	10	0.0184617	7.4e-05	7.4e-05	ID2	EGR1, KMT2A, OGT, BHLHE40, HUWE1
GO:0071013 (GO:0071013)	catalytic step 2 spliceosome	4.540842	10	0.0304022	8.3e-05	3.6e-03		HNRNPH1, PNN, PRPF4B, SRRM1, SRRM2
GO:0045893 (GO:0045893)	positive regulation of transcription, DNA-templated	2.932256	3	0.0066225	8.4e-05	6.5e-04	CEBPB, ID2, YWHAH	KLF6, DDIT3, EGR1, ELF1, EP300, LRP6, KMT2A, RLF, RREB1, FZD4, MED13, MAP3K2, ZNF318, PHIP, KDM3A, EPC1
GO:0051087 (GO:0051087)	chaperone binding	2.796203	6	0.0091159	8.6e-05	1.1e-02		FN1, HSPA8, SACS
GO:0001227 (GO:0001227)	DNA-binding transcription repressor activity, RNA polymerase II-specific	3.199096	10	0.0119760	1.1e-04	2.4e-04	CEBPB, NR3C1, TGIF1	PROX1, TBX3, ZNF217, BHLHE40
GO:0042026 (GO:0042026)	protein refolding	10.119590	10	0.0119760	1.3e-04	2.4e-03		HSPA1A, HSPA8
GO:0097322 (GO:0097322)	7SK snRNA binding	17.709283	9	0.0061728	1.6e-04	3.9e-02		CCNT1, CCNT2
GO:0008013 (GO:0008013)	beta-catenin binding	4.293160	9	0.0061350	3.8e-04	3.7e-02		EP300, DLG5, KANK1, AMER1
GO:0070059 (GO:0070059)	intrinsic apoptotic signaling pathway in response to endoplasmic reticulum stress	11.806189	10	0.0121230	4.6e-04	2.7e-03	CEBPB, TRIB3	DDIT3, PPP1R15A
GO:0008022 (GO:0008022)	protein C-terminus binding	3.785190	10	0.0254176	5.3e-04	4.6e-03		EP300, FN1, MKI67, POLR2A, ATXN2, CEP250, PRRC2C
GO:0034774 (GO:0034774)	secretory granule lumen	2.507509	10	0.0061539	5.4e-04	8.5e-04		SERPINA3, C3, HSPA8, HUWE1
GO:0005159 (GO:0005159)	insulin-like growth factor receptor binding	11.806189	1	0.0061350	9.8e-04	9.8e-04	YWHAH	PIK3R1
GO:0043484 (GO:0043484)	regulation of RNA splicing	12.213299	10	0.0153944	1.4e-03	3.8e-03		CLK1, HNRNPH1, AKAP17A, CLK4, AHNAK
GO:0031625 (GO:0031625)	ubiquitin protein ligase binding	2.351440	10	0.0321627	1.5e-03	4.7e-03	TRIB3, MAP1LC3B	HSPA1A, HSPA8, POLR2A, TRAF6, FZD4, HERC2
GO:0034063 (GO:0034063)	stress granule assembly	11.184811	10	0.0125020	1.6e-03	1.6e-03		DYNC1H1, ATXN2, PRRC2C
GO:0030838 (GO:0030838)	positive regulation of actin filament polymerization	4.722476	5	0.0059880	2.6e-03	2.6e-03	PFN2, BAIAP2L1	
GO:0031398 (GO:0031398)	positive regulation of protein ubiquitination	4.427321	1	0.0103093	2.8e-03	2.8e-03		HUWE1, CDK5RAP3, AMER1
GO:0008134 (GO:0008134)	transcription factor binding	1.717264	7	0.0061350	2.8e-03	4.5e-03		CCNT1, DDIT3, EP300, PIK3R1
GO:0031072 (GO:0031072)	heat shock protein binding	3.728270	8	0.0060615	2.9e-03	2.9e-03		HSPA1A, HSPA8
GO:0006357 (GO:0006357)	regulation of transcription by RNA polymerase II	3.700447	1	0.0058480	3.6e-03	3.6e-03	CEBPB	CHD2, DDIT3, TBX3, OGT,

								CREBZF, TTC21B
GO:0061630 (GO:0061630)	ubiquitin protein ligase activity	2.833485	4	0.0065792	3.7e-03	3.7e-03	RNF41	TRAF6, HERC2, HUWE1, UBR4
GO:0000977 (GO:0000977)	RNA polymerase II transcription regulatory region sequence-specific DNA binding	2.213660	2	0.0062352	3.9e-03	9.9e-03	CEBPB, NR3C1	EGR1, RREB1
GO:0070577 (GO:0070577)	lysine-acetylated histone binding	10.119590	1	0.0059880	5.1e-03	5.1e-03		KMT2A, BAZ2A, PHIP
GO:0003677 (GO:0003677)	DNA binding	2.770167	7	0.0063291	5.7e-03	1.0e-02	CEBPB, ANKRD1	SERPINA3, CCNT1, CHD2, DDIT3, EGR1, EP300, POU2F1, PROX1, HUWE1, RBM6, TET1, ARID5B
GO:0033120 (GO:0033120)	positive regulation of RNA splicing	16.347031	9	0.0065359	5.9e-03	1.2e-02		HSPA1A, PIK3R1, POLR2A
GO:1900028 (GO:1900028)	negative regulation of ruffle assembly	28.334853	1	0.0058480	5.9e-03	5.9e-03	PFN2	KANK1
GO:0051082 (GO:0051082)	unfolded protein binding	2.724505	8	0.0060615	7.6e-03	7.6e-03		HSPA1A, HSPA8
GO:0032956 (GO:0032956)	regulation of actin cytoskeleton organization	4.829805	2	0.0059180	8.4e-03	1.1e-02		LRP1, TAOK2, RICTOR
GO:0055131 (GO:0055131)	C3HC4-type RING finger domain binding	23.612378	10	0.0598802	8.9e-03	2.1e-02		HSPA1A, HSPA8
GO:0030056 (GO:0030056)	hemidesmosome	11.806189	9	0.0061350	8.9e-03	1.1e-02		PLEC
GO:0030011 (GO:0030011)	maintenance of cell polarity	11.806189	4	0.0064325	8.9e-03	3.8e-02		DLG5
GO:0000423 (GO:0000423)	mitophagy	11.806189	2	0.0060104	8.9e-03	1.7e-02	MAP1LC3B	
GO:0097718 (GO:0097718)	disordered domain specific binding	6.159751	10	0.0061539	9.0e-03	1.2e-02		FN1, HSPA1A
GO:0016575 (GO:0016575)	histone deacetylation	7.456540	1	0.0058480	9.1e-03	9.1e-03		BAZ2A, MIER3
GO:0009267 (GO:0009267)	cellular response to starvation	5.059795	10	0.0061539	9.5e-03	1.2e-02	KLF10, MAP1LC3B	HSPA8
GO:0004468 (GO:0004468)	lysine N-acetyltransferase activity, acting on acetyl phosphate as donor	28.334853	7	0.0059880	9.5e-03	9.5e-03		EP300, ATAT1
GO:0018393 (GO:0018393)	internal peptidyl-lysine acetylation	14.167427	10	0.0118360	9.5e-03	2.2e-02		EP300
GO:0045815 (GO:0045815)	positive regulation of gene expression, epigenetic	14.167427	7	0.0059880	9.5e-03	9.5e-03		EP300
GO:0018394 (GO:0018394)	peptidyl-lysine acetylation	11.806189	10	0.0177540	1.1e-02	2.1e-02		EP300
GO:0120183 (GO:0120183)	positive regulation of focal adhesion disassembly	23.612378	6	0.0060615	1.1e-02	4.8e-02		PIK3R1, IQSEC1
GO:0007623 (GO:0007623)	circadian rhythm	8.500456	9	0.0065359	1.3e-02	2.7e-02	ID2	EP300, BHLHE40
GO:0061733 (GO:0061733)	peptide-lysine-N-acetyltransferase activity	11.806189	10	0.0118360	1.4e-02	2.1e-02		EP300
GO:0042752 (GO:0042752)	regulation of circadian rhythm	4.885320	10	0.0061539	1.5e-02	1.5e-02	ID2, KLF10	PROX1, BHLHE40
GO:0035567 (GO:0035567)	non-canonical Wnt signaling pathway	10.119590	9	0.0061350	1.6e-02	2.9e-02		FZD4
GO:0019773 (GO:0019773)	proteasome core complex, alpha-subunit complex	8.854642	9	0.0119760	1.7e-02	2.7e-02	PSMA7	
GO:0005577 (GO:0005577)	fibrinogen complex	17.709283	4	0.0062620	1.7e-02	2.7e-02		FGL1, FN1
GO:0042393 (GO:0042393)	histone binding	4.071100	3	0.0059880	1.7e-02	2.6e-02	ASF1A	ATRX, CHD2, BAZ2A, MYSM1
GO:0006355 (GO:0006355)	regulation of transcription, DNA-templated	3.079875	1	0.0058480	1.7e-02	1.7e-02	CEBPB, NR3C1	ATRX, DDIT3, IGSF1, RREB1, ZNF217, RBAK
GO:0005813 (/Users/nstda/Desktop/DES_13Nov/GO/term_visualizations/centrosome.png)	centrosome (/Users/nstda/Desktop/DES_13Nov/GO/term_visualizations/centrosome.png)	1.472365	6	0.0060615	1.7e-02	1.7e-02		DYNC1H1, DYNC1LI2, HSPA1A, PCNT, ZNF12, CEP250, UBR4, CAPRIN2, CDK5RAP3
GO:0031588 (GO:0031588)	nucleotide-activated protein kinase complex	11.806189	1	0.0058480	1.7e-02	1.7e-02		PRKAB2
GO:0034976 (GO:0034976)	response to endoplasmic reticulum stress	7.327979	10	0.0118360	1.7e-02	4.0e-02	CEBPB, TRIB3	DDIT3, PIK3R1, PPP1R15A, CDK5RAP3
GO:0016604 (GO:0016604)	nuclear body	2.434266	3	0.0063291	2.1e-02	2.1e-02		ATRX, HIVEP1, MKI67, RREB1, SCN1A, BHLHE40, RAPGEF5, NUFIP2, AMER1, RNF169
GO:0150012 (GO:0150012)	positive regulation of neuron projection arborization	8.854642	9	0.0061350	2.1e-02	3.9e-02		FZD4
GO:0004129 (GO:0004129)	cytochrome-c oxidase activity	7.870793	6	0.0060615	2.1e-02	2.7e-02		COX2
GO:0000398 (GO:0000398)	mRNA splicing, via spliceosome	1.507173	1	0.0061350	2.2e-02	2.2e-02		DDX39B, SRRM2
GO:0120162 (GO:0120162)	positive regulation of cold-induced thermogenesis	3.728270	6	0.0062320	2.2e-02	3.3e-02	CEBPB	DYNC1H1, PRKAB2, OGT, KDM3A
GO:0016573 (GO:0016573)	histone acetylation	10.119590	10	0.0061539	2.4e-02	2.9e-02		EP300
GO:0045664 (GO:0045664)	regulation of neuron differentiation	20.239181	1	0.0063291	2.4e-02	2.4e-02	ID2, YWHAH	
GO:0001889 (GO:0001889)	liver development	18.889902	8	0.0062510	2.6e-02	4.4e-02		PROX1, TGFBR3, CDK5RAP3, ARID5B
GO:0033962 (GO:0033962)	P-body assembly	17.709283	3	0.0061350	2.7e-02	2.7e-02		DYNC1H1, ATXN2
GO:0000346 (GO:0000346)	transcription export complex	7.083713	1	0.0063291	2.7e-02	2.7e-02		DDX39B
GO:0005078 (GO:0005078)	MAP-kinase scaffold activity	7.083713	1	0.0061350	2.7e-02	2.7e-02		MAPK8IP3
GO:0006396 (GO:0006396)	RNA processing	5.592405	8	0.0063544	2.7e-02	4.3e-02		HNRNPH1, RBM39, RBM6
GO:0051091 (GO:0051091)	positive regulation of DNA-binding transcription factor activity	4.521519	8	0.0061539	3.2e-02	3.2e-02		DDIT3, EP300, LRP6, TRAF6, FZD4, ARID5B
GO:0006110 (GO:0006110)	regulation of glycolytic process	12.879479	10	0.0118360	3.3e-02	3.3e-02		EP300, OGT
GO:0030837 (GO:0030837)	negative regulation of actin filament polymerization	12.879479	1	0.0058480	3.3e-02	3.3e-02	PFN2	KANK1
GO:0043034 (/Users/nstda/Desktop/DES_13Nov/GO/term_visualizations/costamere.png)	costamere (/Users/nstda/Desktop/DES_13Nov/GO/term_visualizations/costamere.png)	12.879479	1	0.0103093	3.3e-02	3.3e-02		PLEC, AHNAK
GO:0005874 (/Users/nstda/Desktop/DES_13Nov/GO/term_visualizations/microtubule.png)	microtubule (/Users/nstda/Desktop/DES_13Nov/GO/term_visualizations/microtubule.png)	2.500134	1	0.0058480	3.4e-02	3.4e-02		DYNC1H1, MACF1, CDK5RAP3
GO:0015629 (GO:0015629)	actin cytoskeleton	2.414902	1	0.0058480	3.5e-02	3.5e-02	BAIAP2L1	TPM2, TAOK2, ONECUT2, KLHL14, AHNAK

GO:0061629 (GO:0061629)	RNA polymerase II-specific DNA-binding transcription factor binding	4.047836	2	0.0059915	3.5e-02	4.8e-02	ID2, ANKRD1	EP300, TBX3, BHLHE40, TCERG1
GO:0070062 (GO:0070062)	extracellular exosome	1.914517	5	0.0059880	3.6e-02	3.6e-02		FN1, HSPA8, AHNAK
GO:0048024 (GO:0048024)	regulation of mRNA splicing, via spliceosome	5.903094	3	0.0065359	3.9e-02	3.9e-02		RBM39
GO:0043984 (GO:0043984)	histone H4-K16 acetylation	11.184811	4	0.0060804	3.9e-02	4.8e-02		KMT2A, OGT, MSL2
GO:1904813 (GO:1904813)	ficolin-1-rich granule lumen	1.713802	5	0.0061728	4.1e-02	4.9e-02		HSPA1A, HSPA8, HUWE1
GO:0010884 (GO:0010884)	positive regulation of lipid storage	12.879479	1	0.0058480	4.2e-02	4.2e-02		APOB, C3
GO:0060100 (GO:0060100)	positive regulation of phagocytosis, engulfment	15.741585	1	0.0066225	4.2e-02	4.2e-02	F2RL1	C3
GO:0032206 (GO:0032206)	positive regulation of telomere maintenance	7.870793	1	0.0066225	4.2e-02	4.2e-02		ATRX
GO:0010494 (GO:0010494)	cytoplasmic stress granule	3.863844	10	0.0061539	4.2e-02	4.2e-02	ZFAND1	ATXN2, NUFIP2
GO:0070628 (GO:0070628)	proteasome binding	14.167427	8	0.0060615	4.3e-02	4.3e-02	ZFAND1	SACS
GO:0090166 (GO:0090166)	Golgi disassembly	11.806189	7	0.0061728	4.3e-02	4.8e-02		GBF1
GO:0015630 (GO:0015630)	microtubule cytoskeleton	1.180619	2	0.0060804	4.5e-02	4.5e-02		RBM39, NINL
GO:1901029 (GO:1901029)	negative regulation of mitochondrial outer membrane permeabilization involved in apoptotic signaling pathway	20.239181	1	0.0103093	4.7e-02	4.7e-02	SLC35F6	HSPA1A
GO:0051015 (GO:0051015)	actin filament binding	2.236962	1	0.0066225	4.7e-02	4.7e-02	MISP	TPM2, MACF1
