# Supplementary material for: Transcriptomic screening of novel targets of sericin in human hepatocellular carcinoma cells
Source: Sci Rep. 2024 Mar 5;14:5455. doi: 10.1038/s41598-024-56179-y (PMC10914811; doi:10.1038/s41598-024-56179-y)
Supplement: Supplementary file 8 — Supplementary Table S4. [file 41598_2024_56179_MOESM8_ESM.pdf]

GO-Analysis (0.125 mg/ mL vs 1 mg/ mL)

ID	Term_Description	Fold_Enrichment	occurrence	support	lowest_p	highest_p	Up_regulated	Down_regulated
GO:0042026	protein refolding	221.90816	8	0.2353846	4.2e-06	2.9e-02		HSPA1A, HSPA8
GO:0001664	G protein-coupled receptor binding	88.76327	5	0.0454545	7.6e-05	7.6e-05		HSPA1A, HSPA8
GO:0031072	heat shock protein binding	81.75564	5	0.0454545	9.8e-05	9.8e-05		HSPA1A, HSPA8
GO:0051082	unfolded protein binding	59.74451	1	0.0400000	6.4e-04	6.4e-04		HSPA1A, HSPA8
GO:0031625	ubiquitin protein ligase binding	19.33640	8	0.1076923	1.1e-03	2.7e-02	TRIB3	HSPA1A, HSPA8
GO:0043202	lysosomal lumen	36.54958	2	0.0476190	1.1e-03	1.1e-03		APOB, HSPA8
GO:0055131	C3HC4-type RING finger domain binding	517.78571	8	0.2390476	1.9e-03	4.8e-03		HSPA1A, HSPA8
GO:1904813	ficolin-1-rich granule lumen	25.05415	4	0.0465368	3.6e-03	3.6e-03		HSPA1A, HSPA8
GO:0031396	regulation of protein ubiquitination	155.33571	2	0.0476190	5.7e-03	5.7e-03		HSPA1A
GO:0051787	misfolded protein binding	129.44643	6	0.0454545	8.4e-03	1.4e-02		HSPA1A
GO:0003714	transcription corepressor activity	37.88676	3	0.0400000	8.8e-03	8.8e-03	TRIB3, AJUBA	HSPA1A
GO:0031146	SCF-dependent proteasomal ubiquitin-dependent protein catabolic process	73.96939	6	0.0476190	1.3e-02	1.3e-02	FBXO2	
GO:0101031	chaperone complex	91.37395	5	0.0454545	1.7e-02	1.7e-02		HSPA8
GO:0070059	intrinsic apoptotic signaling pathway in response to endoplasmic reticulum stress	129.44643	6	0.0465368	1.7e-02	3.5e-02	CEBPB, TRIB3	
GO:0051131	chaperone-mediated protein complex assembly	86.29762	2	0.0476190	1.9e-02	1.9e-02		HSPA1A
GO:0071682	endocytic vesicle lumen	86.29762	2	0.0476190	1.9e-02	1.9e-02		APOB
GO:0005524	ATP binding	13.93145	2	0.0476190	2.1e-02	2.1e-02		HSPA1A, HSPA8
GO:0046034	ATP metabolic process	163.51128	7	0.1904762	2.2e-02	2.2e-02		HSPA1A, HSPA8
GO:0019005	SCF ubiquitin ligase complex	53.56404	6	0.0465368	2.6e-02	2.6e-02	FBXO2	
GO:0097718	disordered domain specific binding	67.53727	4	0.0726190	3.2e-02	3.2e-02		HSPA1A
GO:0045599	negative regulation of fat cell differentiation	43.14881	1	0.0400000	4.0e-02	4.0e-02	TRIB3	
GO:0034605	cellular response to heat	57.53175	5	0.0454545	4.4e-02	4.4e-02		HSPA1A
