# Supplementary material for: Transcriptomic screening of novel targets of sericin in human hepatocellular carcinoma cells
Source: Sci Rep. 2024 Mar 5;14:5455. doi: 10.1038/s41598-024-56179-y (PMC10914811; doi:10.1038/s41598-024-56179-y)
Supplement: Supplementary file 9 — Supplementary Table S5. [file 41598_2024_56179_MOESM9_ESM.pdf]

KEGG and Reactome Enriched Terms (0.125 mg/ mL vs untreated)

KEGG

ID	Term_Description	Fold_Enrichment	occurrence	support	lowest_p	highest_p	Up_regulated	Down_regulated
hsa04610	Complement and coagulation cascades	12.628920	7	0.0061728	7.3e-05	7.3e-05	SERPINE1	F5, SERPINA5, A2M
hsa04380	Osteoclast differentiation	4.277537	9	0.0114286	2.4e-04	3.2e-02	SQSTM1	IL1R1
hsa05131	Shigellosis	2.455623	8	0.0076500	1.3e-03	1.1e-02	SQSTM1	IL1R1
hsa05418	Fluid shear stress and atherosclerosis	7.687169	10	0.0134325	1.6e-03	1.6e-03	SQSTM1	ITGAV, IL1R1, VEGFA
hsa04210	Apoptosis	3.928997	5	0.0054645	2.7e-03	2.7e-03	TUBA1B	DAB2IP
hsa04060	Cytokine-cytokine receptor interaction	1.867657	10	0.0059436	2.9e-03	2.9e-03		LIFR, IL1R1
hsa04510	Focal adhesion	2.678862	10	0.0059436	4.8e-03	4.8e-03		ITGAV, VEGFA
hsa05014	Amyotrophic lateral sclerosis	1.560043	10	0.0067162	4.9e-03	2.4e-02	SQSTM1, TUBA1B	

ID	Term_Description	Fold_Enrichment	occurrence	support	lowest_p	highest_p	Up_regulated	Down_regulated
hsa05205	Proteoglycans in cancer	3.919320	10	0.0169748	5.2e-03	5.2e-03		ERBB3, VEGFA, ITGAV
hsa04392	Hippo signaling pathway - multiple species	9.471690	10	0.0107904	5.3e-03	7.9e-03		AJUBA

Reactome

ID	Term_Description	Fold_Enrichment	occurrence	support	lowest_p	highest_p	Up_regulated	Down_regulated
R-HSA-9020702	Interleukin-1 signaling	5.251630	6	0.0055453	0.00074	0.00074	SQSTM1	IL1R1
R-HSA-2219530	Constitutive Signaling by Aberrant PI3K in Cancer	6.800188	8	0.0058313	0.00084	0.00084		ERBB3, KLB
R-HSA-5661231	Metallothioneins bind metals	79.562195	10	0.0116627	0.00109	0.00363	MT1E, MT1G, MT2A	
R-HSA-1306955	GRB7 events in ERBB2 signaling	53.041463	9	0.0058824	0.00121	0.00121		ERBB3
R-HSA-5660526	Response to metal ions	61.201689	10	0.0116627	0.00189	0.00630	MT1E, MT1G, MT2A	

ID	Term_Description	Fold_Enrichment	occurrence	support	lowest_p	highest_p	Up_regulated	Down_regulated
R-HSA-446652	Interleukin-1 family signaling	3.843584	6	0.0055453	0.00190	0.00190	SQSTM1	IL1R1
R-HSA-2219528	PI3K/AKT Signaling in Cancer	5.051568	8	0.0058313	0.00208	0.00208		ERBB3, KLB
R-HSA-6811558	PI5P, PP2A and IER3 Regulate PI3K/AKT Signaling	5.003912	8	0.0058313	0.00214	0.00214		ERBB3, KLB
R-HSA-199418	Negative regulation of the PI3K/AKT network	4.693935	8	0.0058313	0.00259	0.00259		ERBB3, KLB
R-HSA-1234158	Regulation of gene expression by Hypoxia-inducible Factor	24.109756	8	0.0058313	0.00267	0.00267		VEGFA
R-HSA-75205	Dissolution of Fibrin Clot	20.400563	8	0.0058313	0.00378	0.00378	SERPINE1	
R-HSA-209560	NF-kB is activated and signals survival	20.400563	7	0.0104167	0.00378	0.00944	SQSTM1	
R-HSA-209543	p75NTR recruits signalling complexes	20.400563	7	0.0104167	0.00378	0.00944	SQSTM1	

ID	Term_Description	Fold_Enrichment	occurrence	support	lowest_p	highest_p	Up_regulated	Down_regulated
R-HSA-3214842	HDMs demethylate histones	34.592259	8	0.0058313	0.00447	0.00636		ARID5B, KDM6B, KDM7A
R-HSA-210990	PECAM1 interactions	44.201220	9	0.0117647	0.00533	0.00533		INPP5D, ITGAV
R-HSA-205043	NRIF signals cell death from the nucleus	16.575457	7	0.0104167	0.00581	0.01452	SQSTM1	
R-HSA-193639	p75NTR signals via NF-kB	16.575457	7	0.0104167	0.00581	0.01452	SQSTM1	
R-HSA-140837	Intrinsic Pathway of Fibrin Clot Formation	23.061506	5	0.0052083	0.00613	0.00613		A2M, SERPINA5
R-HSA-9664873	Pexophagy	24.109756	7	0.0057803	0.00666	0.00666	SQSTM1	
R-HSA-6785807	Interleukin-4 and Interleukin-13 signaling	4.778510	10	0.0058998	0.00853	0.01359		BCL6, VEGFA
