# Supplementary material for: Transcriptomic screening of novel targets of sericin in human hepatocellular carcinoma cells
Source: Sci Rep. 2024 Mar 5;14:5455. doi: 10.1038/s41598-024-56179-y (PMC10914811; doi:10.1038/s41598-024-56179-y)
Supplement: Supplementary file 10 — Supplementary Table S6. [file 41598_2024_56179_MOESM10_ESM.pdf]

KEGG and Reactome Enriched Terms (1 mg/ mL vs untreated)

KEGG

ID	Term_Description	Fold_Enrichment	occurrence	support	lowest_p	highest_p	Up_regulated	Down_regulated
hsa04010	MAPK signaling pathway	2.1908392	10	0.0121636	3.6e-07	2.0e-03		PPP3CA, FGFR1, TRAF6, MAP3K2, TAOK2, MAPK8IP3, DDIT3, HSPA8, HSPA1A
hsa03040	Spliceosome	3.0143461	10	0.0330642	3.5e-06	1.6e-03		DDX39B, RBM25, TCERG1, HSPA8, HSPA1A, SRSF5
hsa05417	Lipid and atherosclerosis	2.9791318	10	0.0520249	5.4e-05	1.6e-03		APOB, PIK3R1, POU2F1, HSPA8, HSPA1A, TRAF6, DDIT3, ABCA1, PPP3CA
hsa04068	FoxO signaling pathway	2.2136604	10	0.0179647	7.7e-05	7.7e-05		PRKAB2, EP300, PIK3R1, BCL6
hsa05134	Legionellosis	6.2137836	10	0.0213984	1.2e-04	6.7e-04		C3, CLK4, CLK1, HSPA8, HSPA1A

ID	Term_Description	Fold_Enrichment	occurrence	support	lowest_p	highest_p	Up_regulated	Down_regulated
hsa04141	Protein processing in endoplasmic reticulum	2.6235975	10	0.0268678	2.0e-04	2.0e-04	FBXO2	RRBP1, HSPA8, HSPA1A, PPP1R15A, DDIT3
hsa04962	Vasopressin-regulated water reabsorption	3.2198697	7	0.0062500	3.0e-04	3.7e-02		DYNC1H1, DYNC1LI2
hsa05202	Transcriptional misregulation in cancer	2.8829066	10	0.0232558	3.9e-04	1.2e-02	ID2, CEBPB	KMT2A, CCNT1, CCNT2, BCL6, DDIT3
hsa05020	Prion disease	2.4806778	10	0.0175967	4.2e-04	2.0e-02	PSMA7	DDIT3, ITPR2, PPP3CA, COX2, HSPA8, HSPA1A, PIK3R1, EGR1
hsa05100	Bacterial invasion of epithelial cells	2.0532502	9	0.0059172	5.6e-04	5.6e-04		PIK3R1, FN1

Reactome

ID	Term_Description	Fold_Enrichment	occurrence	support	lowest_p	highest_p	Up_regulated	Down_regulated
R-HSA-9663891	Selective autophagy	9.290116	10	0.0344181	7.0e-08	1.3e-04	MAP1LC3B, RPS27A, TOMM5	DYNC1H1, DYNC1LI2, HSPA8, PCNT, PRKAB2
R-HSA-72163	mRNA Splicing - Major Pathway	2.374429	10	0.0295564	3.1e-07	2.0e-03		HNRNPH1, HSPA8, POLR2A, SRRM1, SRRM2, SRSF5
R-HSA-72172	mRNA Splicing	2.236962	10	0.0272822	4.7e-07	2.7e-03		HNRNPH1, HSPA8, POLR2A, SRRM1, SRRM2, SRSF5
R-HSA-3371497	HSP90 chaperone cycle for steroid hormone receptors (SHR) in the presence of ligand	9.320676	10	0.0474607	6.1e-07	3.2e-06	NR3C1	DYNC1H1, DYNC1LI2, HSPA1A, HSPA8
R-HSA-380259	Loss of Nlp from mitotic centrosomes	4.166890	8	0.0065153	6.2e-07	1.6e-02		CEP250, DYNC1H1, NINL, PCNT
R-HSA-380284	Loss of proteins required for interphase microtubule	4.166890	8	0.0065153	6.2e-07	1.6e-02		CEP250, DYNC1H1, NINL, PCNT

ID	Term_Description	Fold_Enrichment	occurrence	support	lowest_p	highest_p	Up_regulated	Down_regulated
	organization from the centrosome							
R-HSA-8854518	AURKA Activation by TPX2	3.990824	8	0.0065153	7.7e-07	1.8e-02		CEP250, DYNC1H1, NINL, PCNT
R-HSA-380320	Recruitment of NuMA to mitotic centrosomes	3.586690	8	0.0065153	1.3e-06	2.5e-02		CEP250, DYNC1H1, NINL, PCNT
R-HSA-380287	Centrosome maturation	3.541857	8	0.0065153	1.4e-06	2.6e-02		CEP250, DYNC1H1, NINL, PCNT
R-HSA-380270	Recruitment of mitotic centrosome proteins and complexes	3.541857	8	0.0065153	1.4e-06	2.6e-02		CEP250, DYNC1H1, NINL, PCNT
R-HSA-1632852	Macroautophagy	4.885320	10	0.0264700	1.9e-06	3.3e-03	MAP1LC3B, RPS27A, TOMM5	DYNC1H1, DYNC1LI2, HSPA8, PCNT, PRKAB2
R-HSA-2565942	Regulation of PLK1 Activity at G2/M Transition	4.118438	8	0.0065153	2.1e-06	3.2e-02	RPS27A	CEP250, DYNC1H1, NINL, PCNT

ID	Term_Description	Fold_Enrichment	occurrence	support	lowest_p	highest_p	Up_regulated	Down_regulated
R-HSA-72203	Processing of Capped Intron-Containing Pre-mRNA	2.057510	10	0.0354677	2.5e-06	2.9e-04		DDX39B, HNRNPH1, HSPA8, POLR2A, SRRM1, SRRM2, SRSF5
R-HSA-5620912	Anchoring of the basal body to the plasma membrane	2.982616	8	0.0065153	3.4e-06	4.3e-02		CEP250, DYNC1H1, NINL, PCNT
R-HSA-9612973	Autophagy	4.325932	10	0.0256790	3.5e-06	6.0e-03	MAP1LC3B, RPS27A, TOMM5	DYNC1H1, DYNC1LI2, HSPA8, PCNT, PRKAB2
R-HSA-5663202	Diseases of signal transduction by growth factor receptors and second messengers	2.840168	10	0.0177521	1.2e-05	2.5e-03	CEBPB, PSMA7, RPS27A	AMER1, BIRC6, EP300, FGFR1, FN1, FZD4, GOLGB1, KANK1, KDM7A, LRP6, PIK3R1, POLR2A, RICTOR, RRBP1
R-HSA-5617833	Cilium Assembly	2.801469	2	0.0058010	1.6e-05	7.9e-05		ATAT1, CEP250, DYNC1H1, GBF1, NINL, PCNT, TTC21B

ID	Term_Description	Fold_Enrichment	occurrence	support	lowest_p	highest_p	Up_regulated	Down_regulated
R-HSA-68877	Mitotic Prometaphase	1.935441	7	0.0061728	1.9e-05	6.2e-03		CEP250, DYNC1H1, DYNC1LI2, NINL, PCNT
R-HSA-9648895	Response of EIF2AK1 (HRI) to heme deficiency	18.889902	10	0.0171630	2.5e-05	2.3e-04	CEBPB, TRIB3	DDIT3, PPP1R15A
R-HSA-75035	Chk1/Chk2(Cds1) mediated inactivation of Cyclin B:Cdk1 complex	10.898020	10	0.0123457	2.7e-05	5.6e-04	SFN, YWHAH	
