# Supplementary material for: Transcriptomic screening of novel targets of sericin in human hepatocellular carcinoma cells
Source: Sci Rep. 2024 Mar 5;14:5455. doi: 10.1038/s41598-024-56179-y (PMC10914811; doi:10.1038/s41598-024-56179-y)
Supplement: Supplementary file 11 — Supplementary Table S7. [file 41598_2024_56179_MOESM11_ESM.pdf]

KEGG and Reactome Enriched Terms (0.125 mg/mL vs 1 mg/mL)

KEGG (Different)

ID	Term_Description	Fold_Enrichment	occurrence	support	lowest_p	highest_p	Up_regulated	Down_regulated
hsa04141	Protein processing in endoplasmic reticulum	28.76587	10	0.0952381	9.8e-07	9.8e-07	FBXO2	HSPA8, HSPA1A
hsa05417	Lipid and atherosclerosis	21.77603	10	0.0869565	3.0e-06	1.2e-03		APOB, HSPA8, HSPA1A
hsa04612	Antigen processing and presentation	41.98263	10	0.1904762	5.0e-05	2.3e-02		HSPA8, HSPA1A
hsa04915	Estrogen signaling pathway	22.67675	8	0.0476190	3.2e-04	2.8e-03		HSPA8, HSPA1A
hsa05162	Measles	22.51242	6	0.0476190	3.3e-04	3.3e-04		HSPA8, HSPA1A
hsa03040	Spliceosome	22.03343	3	0.0434783	3.5e-04	1.7e-03		HSPA8, HSPA1A
hsa05020	Prion disease	18.13257	10	0.0500000	2.1e-03	2.9e-02		COX2, HSPA8, HSPA1A
hsa04010	MAPK signaling pathway	10.67599	10	0.0869565	3.1e-03	4.2e-02		HSPA8, HSPA1A

ID	Term_Description	Fold_Enrichment	occurrence	support	lowest_p	highest_p	Up_regulated	Down_regulated
hsa05134	Legionellosis	54.50376	10	0.2039337	1.3e-02	1.3e-02		HSPA8, HSPA1A
hsa04144	Endocytosis	12.89093	2	0.0434783	1.5e-02	1.5e-02		HSPA8, HSPA1A

Reactome (Different)

ID	Term_Description	Fold_Enrichment	occurrence	support	lowest_p	highest_p	Up_regulated	Down_regulated
R-HSA-3371568	Attenuation phase	119.48901	8	0.2287582	1.2e-05	0.01574		HSPA1A, HSPA8
R-HSA-3371571	HSF1-dependent transactivation	86.29762	8	0.1281818	3.2e-05	0.03049		HSPA1A, HSPA8
R-HSA-3371497	HSP90 chaperone cycle for steroid hormone receptors (SHR) in the presence of ligand	81.75564	8	0.1281818	3.8e-05	0.03401		HSPA1A, HSPA8
R-HSA-450408	AUF1 (hnRNP D0) binds and destabilizes mRNA	57.53175	1	0.0285714	1.1e-04	0.00011		HSPA1A, HSPA8

ID	Term_Description	Fold_Enrichment	occurrence	support	lowest_p	highest_p	Up_regulated	Down_regulated
R-HSA-450531	Regulation of mRNA stability by proteins that bind AU-rich elements	35.30357	1	0.0285714	4.9e-04	0.00049		HSPA1A, HSPA8
R-HSA-3371556	Cellular response to heat stress	32.02798	5	0.0454545	6.6e-04	0.00066		HSPA1A, HSPA8
R-HSA-3000484	Scavenging by Class F Receptors	258.89286	5	0.0454545	7.3e-04	0.00073		APOB
R-HSA-9725371	Nuclear events stimulated by ALK signaling in cancer	81.75564	5	0.0370370	4.1e-03	0.00414	CEBPB	
R-HSA-9648895	Response of EIF2AK1 (HRI) to heme deficiency	207.11429	7	0.0800000	5.1e-03	0.00509	CEBPB, TRIB3	
R-HSA-9613829	Chaperone Mediated Autophagy	73.96939	5	0.0454545	1.0e-02	0.01017		HSPA8
R-HSA-3371511	HSF1 activation	53.56404	6	0.0412458	2.0e-02	0.03273		HSPA1A
R-HSA-389356	CD28 co-stimulation	47.07143	4	0.0357599	2.6e-02	0.04255	TRIB3	

ID	Term_Description	Fold_Enrichment	occurrence	support	lowest_p	highest_p	Up_regulated	Down_regulated
R-HSA-432720	Lysosome Vesicle Biogenesis	44.38163	7	0.0400000	2.9e-02	0.02879		HSPA8
R-HSA-9725370	Signaling by ALK fusions and activated point mutants	29.30863	5	0.0370370	3.3e-02	0.03336	CEBPB	
R-HSA-9700206	Signaling by ALK in cancer	29.30863	5	0.0370370	3.3e-02	0.03336	CEBPB	
R-HSA-109704	PI3K Cascade	35.30357	1	0.0285714	4.6e-02	0.04575	TRIB3	

Reactome (Both)

ID	Term_Description	Fold_Enrichment	occurrence	support	lowest_p	highest_p	Up_regulated	Down_regulated
R-HSA-5675482	Regulation of necroptotic cell death	20.267474	10	0.0109890	0.0197	0.0197		OGT
R-HSA-5213460	RIPK1-mediated regulated necrosis	19.591892	10	0.0109890	0.0211	0.0211		OGT
