# Supplementary material for: Transcriptomic screening of novel targets of sericin in human hepatocellular carcinoma cells
Source: Sci Rep. 2024 Mar 5;14:5455. doi: 10.1038/s41598-024-56179-y (PMC10914811; doi:10.1038/s41598-024-56179-y)
Supplement: Supplementary file 13 — Supplementary Table S9. [file 41598_2024_56179_MOESM13_ESM.docx]

**Supplementary Table S9**

**The qPCR validation for all genes.**

| Log fold change | 0.125 mg/mL | | | 1 mg/mL | | |
| --- | --- | --- | --- | --- | --- | --- |
| Gene | RNAseq | qPCR | Transcription Activation | RNAseq | qPCR | Transcription Activation |
| *APOB* | -0.0494 | -0.7223 | - | -0.3673 | -2.0366 | - |
| *ARID5B* | -0.6092 | -3.1805 | - | -0.4218 | -2.1833 | - |
| *MT1E* | 0.5104 | 4.7210 | - | 0.2903 | 2.6090 | - |
| *HSPA8* | 0.1965 | -2.2001 | - | -0.2941 | -0.8913 | - |
| *NFE2L2* | -0.2011 | 0.7409 | 1.8547 | 0.1374 | 0.5470 | 3.1467 |
| *STAT1* | 0.0678 | -2.7397 | -1.777 | -0.0383 | -1.4013 | -0.7897 |
| *TFAP2C* | NA | -3.6530 | -1.6349 | NA | -1.3197 | -1.0402 |
| *CEBPA* | 0.1824 | 0.1123 | -2.177 | 0.2897 | -0.0239 | - |
| *CREB1* | -0.0879 | -0.0679 | - | 0.0404 | -0.2126 | -1.3126 |
| *DYNCH1* | NA | -4.8664 | - | NA | -3.3337 | - |
| *HSPA5* | 0.1087 | -0.1034 | - | -0.1192 | -1.6647 | - |
| *KDM6B* | -0.3994 | -0.6089 | - | -0.1909 | -0.7887 | - |
| *MAP1LC3B* | 0.1364 | -2.1729 | - | 0.3185 | -2.5244 | - |
| *FGFR1* | -0.1351 | -2.4627 | - | -0.2858 | -0.8989 | - |
| *A2M* | -0.2888 | -4.1980 | - | -0.2154 | -6.2011 | - |
| *POU2F1* | -0.1473 | -0.5844 | - | -0.3623 | -1.8992 | - |
| *SERPINA5* | -0.2833 | -4.0304 | - | -0.1681 | -1.5712 | - |
| *MT1G* | 0.5667 | 7.0628 | - | 0.2199 | 6.6086 | - |
| *MT2A* | 0.5067 | 1.7088 | - | 0.1521 | 1.7159 | - |
| *KDM7A* | -0.3132 | -2.6295 | - | -0.2703 | -3.6834 | - |
| *OGT* | -0.3018 | -1.0573 | - | -0.3919 | -4.1291 | - |
| *GATA3* | NA | -0.9051 | -1.4012 | NA | -2.1203 | -0.8013 |
