# Supplementary material for: Transcriptomic screening of novel targets of sericin in human hepatocellular carcinoma cells
Source: Sci Rep. 2024 Mar 5;14:5455. doi: 10.1038/s41598-024-56179-y (PMC10914811; doi:10.1038/s41598-024-56179-y)
Supplement: Supplementary file 14 — Supplementary Table S10. [file 41598_2024_56179_MOESM14_ESM.docx]

**Supplementary Table S10**

**Oligonucleotide primers for qRT-PCR**

| **No** | **Gene** | **Primer (5’-3’)** |
| --- | --- | --- |
| 1 | A2M | F: GTCTGTTCTCCTCCAGCTCCT  R: CAGCCCTTCTCAGTGGTCTCA |
| 2 | APOB | F: GACTGTTAGGACACCAGCCC  R: CCCGAAGGCTGAAATGGTCT |
| 3 | ARID5B | F: AAGGTTGCCATTGGTGAAGA  R: GGCGGGCTGTTATTGTTTCAT |
| 4 | BCL6 | F: ATGGAGCATGTTGTGGACACT  R: GGCTGTTGAGGAACTCTTCAC |
| 5 | CEBPA | F: GGACCCTCAGCCTTGTTTGT  R: GCCGACGGAGAGTCTCATTT |
| 6 | CREB1 | F: GTGTGTTACGTGGGGGAGAG  R: GCATCTCCACTCTGCTGGTT |
| 7 | DYNC1H1 | F: TTGCGGCCCTATGGTGAAAT  R: GGATCATCTGCTCCACCTCG |
| 8 | EGR1 | F: AGTGAGCATGACCAACCCAC  R: TGGAAACAGGTAGTCGGGGA |
| 9 | FGFR1 | F: GACTCCGGCCTCTATGCTTG  R: GGAGCTACGGGGTTTGGTTT |
| 10 | GATA3 | F:ACCACAACCACACTCTGGAGGA  R: TCGGTTTCTGGTCTGGATGCCT |
| 11 | HSPA5 | F: TCTTGTTGGTGGCTCGACTC  R: GGTCATGACACCTCCCACAG |
| 12 | HSPA8 | F: CCTTCGTTATTGGAGCCAGG  R: TTGGAGTGGTTCGGTTTCCC |
| 13 | KDM6B | F: CCACCATTGCCAAGTACGC  R: CGCTTAGCATCAGACAAGTCG |
| 14 | KDM7A | F: ACTCAGGCTCCCTTCACCTA  R: TCAGGGACCTCCACCAATTC |
| 15 | KLHL14 | F: CAGCCGATATGATCCTCGATT  R: GTCCAACCGACATGCATAGAA |
| 16 | MAP1LC3B | F: AGCTCCAAGTGAGCACATTCA  R: GGCGGGTTTTGTGAACCTGA |
| 17 | MT1E | F: TGCTTTCCAACTGCCTGACT  R: CCCAGATTCCTGGAGATGGC |
| 18 | MT1G | F: AAAGGGGCATCGGAGAAGTG  R: GCAAAGGGGTCAAGATTGTAGC |
| 19 | MT2A | F: CCCGCTCCCAGATGTAAAGA  R: ATAGCAAACGGTCACGGTCAG |
| 20 | NFE2L2 | F: CACGGTCCACAGCTCATCAT  R: GGCTGGCTGAATTGGGAGAA |
| 21 | OGT | F: GACAGCACAGAACCAACGA  R: TCGATAATGCTCAATTGCCTC |
| 22 | POU2F1 | F: ATGAACAATCCGTCAGAAACCAG  R: GATGGAGATGTCCAAGGAAAGC |
| 23 | SERPINA3 | F: CCTGAGGCAGAGTTGAGAAT  R: GTGTCCCTCGGTCTTGGTTC |
| 24 | SERPINA5 | F: TGTGGCAAAGCAAACGAAGG  R: CAGTCTCCGAGGTCACGTA |
| 25 | SLC16A6 | F: TGGGCGGTAGCTGTTTCATT  R: ACGATTGCTCAGGACTGTGG |
| 26 | STAT1 | F: GCAGGTTCACCAGCTTTATGA  R: AAGATTACGCTTGCTTTTCCT |
| 27 | TCIM | F: TGGCTACCACTTCGACACAG  R: ATCTTGGCTCTCTCCTCTGC |
| 28 | TFAP2C | F: TCAGTCCCTGGAAGATTGTCG  R: CCAGTAACGAGGCATTTAAGCA |
| 29 | TPM2 | F: GAGTTTGCCGAGAGGTCTGTG  R: AGGATTAAAGGGCCTTGAGAG |
| 30 | TRAF6 | F: TCTGTGTCCGTCCTCTACCA  R: AGCACACAAAGAAAGCTGGG |
| 31 | YPEL2 | F: CTGTGGCTTTAAGAGCGTGC  R: TTCGGCAGAAGAACCCTCAC |
