# Supplementary material for: Transcriptomic screening of novel targets of sericin in human hepatocellular carcinoma cells
Source: Sci Rep. 2024 Mar 5;14:5455. doi: 10.1038/s41598-024-56179-y (PMC10914811; doi:10.1038/s41598-024-56179-y)
Supplement: Supplementary file 15 — Supplementary Table S11. [file 41598_2024_56179_MOESM15_ESM.docx]

**Supplementary Table S11**

**Thermal cycle conditions**

| **Gene** | **Cycle step** | **Temperature** | **Time** | **Cycle** |
| --- | --- | --- | --- | --- |
| A2M | Initial Denaturation | 95 °C | 60 second | 1 |
|  | Denaturation | 95 °C | 15 seconds | **39** |
|  | **Annealing** | **67** °C | **60 seconds** |  |
|  | Extension | 72 °C | 20 seconds |  |
| APOB | Initial Denaturation | 95 °C | 60 second | 1 |
|  | Denaturation | 95 °C | 15 seconds | **39** |
|  | **Annealing** | **65.7** | **60 seconds** |  |
|  | Extension | 72 °C | 20 seconds |  |
| ARID5B | Initial Denaturation | 95 °C | 60 second | 1 |
|  | Denaturation | 95 °C | 15 seconds | **39** |
|  | **Annealing** | **60.7 °C** | **60 seconds** |  |
|  | Extension | 72 °C | 20 seconds |  |
| BCL6 | Initial Denaturation | 95 °C | 60 second | 1 |
|  | Denaturation | 95 °C | 15 seconds | **39** |
|  | **Annealing** | **60.7 °C** | **60 seconds** |  |
|  | Extension | 72 °C | 20 seconds |  |
| CREBPA | Initial Denaturation | 95 °C | 60 second | 1 |
|  | Denaturation | 95 °C | 15 seconds | **39** |
|  | **Annealing** | **65 °C** | **60 seconds** |  |
|  | Extension | 72 °C | 20 seconds |  |
| CREB1 | Initial Denaturation | 95 °C | 60 second | 1 |
|  | Denaturation | 95 °C | 15 seconds | **39** |
|  | **Annealing** | **65 °C** | **60 seconds** |  |
|  | Extension | 72 °C | 20 seconds |  |
| DYNC1H1 | Initial Denaturation | 95 °C | 60 second | 1 |
|  | Denaturation | 95 °C | 15 seconds | **39** |
|  | **Annealing** | **65 °C** | **60 seconds** |  |
|  | Extension | 72 °C | 20 seconds |  |
| EGR1 | Initial Denaturation | 95 °C | 60 second | 1 |
|  | Denaturation | 95 °C | 15 seconds | **39** |
|  | **Annealing** | **60.7 °C** | **60 seconds** |  |
|  | Extension | 72 °C | 20 seconds |  |
| FGFR1 | Initial Denaturation | 95 °C | 60 second | 1 |
|  | Denaturation | 95 °C | 15 seconds | **39** |
|  | **Annealing** | **65.7 °C** | **60 seconds** |  |
|  | Extension | 72 °C | 20 seconds |  |
| GATA3 | Initial Denaturation | 95 °C | 60 second | 1 |
|  | Denaturation | 95 °C | 15 seconds | **39** |
|  | **Annealing** | **60 °C** | **60 seconds** |  |
|  | Extension | 72 °C | 20 seconds |  |
| HSPA5 | Initial Denaturation | 95 °C | 60 second | 1 |
|  | Denaturation | 95 °C | 15 seconds | **39** |
|  | **Annealing** | **65 °C** | **60 seconds** |  |
|  | Extension | 72 °C | 20 seconds |  |
| HSPA8 | Initial Denaturation | 95 °C | 60 second | 1 |
|  | Denaturation | 95 °C | 15 seconds | **39** |
|  | **Annealing** | **65.7 °C** | **60 seconds** |  |
|  | Extension | 72 °C | 20 seconds |  |
| KDM6B | Initial Denaturation | 95 °C | 60 second | 1 |
|  | Denaturation | 95 °C | 15 seconds | **39** |
|  | **Annealing** | **65 °C** | **60 seconds** |  |
|  | Extension | 72 °C | 20 seconds |  |
| KDM7A | Initial Denaturation | 95 °C | 60 second | 1 |
|  | Denaturation | 95 °C | 15 seconds | **39** |
|  | **Annealing** | **63.5 °C** | **60 seconds** |  |
|  | Extension | 72 °C | 20 seconds |  |
| KLHL14 | Initial Denaturation | 95 °C | 60 second | 1 |
|  | Denaturation | 95 °C | 15 seconds | **39** |
|  | **Annealing** | **57 °C** | **60 seconds** |  |
|  | Extension | 72 °C | 20 seconds |  |
| MAP1LC3B | Initial Denaturation | 95 °C | 60 second | 1 |
|  | Denaturation | 95 °C | 15 seconds | **39** |
|  | **Annealing** | **65 °C** | **60 seconds** |  |
|  | Extension | 72 °C | 20 seconds |  |
| MT1E | Initial Denaturation | 95 °C | 60 second | 1 |
|  | Denaturation | 95 °C | 15 seconds | **39** |
|  | **Annealing** | **65 °C** | **60 seconds** |  |
|  | Extension | 72 °C | 20 seconds |  |
| MT1G | Initial Denaturation | 95 °C | 60 second | 1 |
|  | Denaturation | 95 °C | 15 seconds | **39** |
|  | **Annealing** | **67 °C** | **60 seconds** |  |
|  | Extension | 72 °C | 20 seconds |  |
| MT2A | Initial Denaturation | 95 °C | 60 second | 1 |
|  | Denaturation | 95 °C | 15 seconds | **39** |
|  | **Annealing** | **67 °C** | **60 seconds** |  |
|  | Extension | 72 °C | 20 seconds |  |
| NFE2L2 | Initial Denaturation | 95 °C | 60 second | 1 |
|  | Denaturation | 95 °C | 15 seconds | **39** |
|  | **Annealing** | **65.7 °C** | **60 seconds** |  |
|  | Extension | 72 °C | 20 seconds |  |
| OGT | Initial Denaturation | 95 °C | 60 second | 1 |
|  | Denaturation | 95 °C | 15 seconds | **39** |
|  | **Annealing** | **65.7 °C** | **60 seconds** |  |
|  | Extension | 72 °C | 20 seconds |  |
| POU2F1 | Initial Denaturation | 95 °C | 60 second | 1 |
|  | Denaturation | 95 °C | 15 seconds | **39** |
|  | **Annealing** | **67 °C** | **60 seconds** |  |
|  | Extension | 72 °C | 20 seconds |  |
| SERPINA3 | Initial Denaturation | 95 °C | 60 second | 1 |
|  | Denaturation | 95 °C | 15 seconds | **39** |
|  | **Annealing** | **60.7 °C** | **60 seconds** |  |
|  | Extension | 72 °C | 20 seconds |  |
| SERPINA5 | Initial Denaturation | 95 °C | 60 second | 1 |
|  | Denaturation | 95 °C | 15 seconds | **39** |
|  | **Annealing** | **67 °C** | **60 seconds** |  |
|  | Extension | 72 °C | 20 seconds |  |
| SLC16A6 | Initial Denaturation | 95 °C | 60 second | 1 |
|  | Denaturation | 95 °C | 15 seconds | **39** |
|  | **Annealing** | **60.7 °C** | **60 seconds** |  |
|  | Extension | 72 °C | 20 seconds |  |
| STAT1 | Initial Denaturation | 95 °C | 60 second | 1 |
|  | Denaturation | 95 °C | 15 seconds | **39** |
|  | **Annealing** | **65.7 °C** | **60 seconds** |  |
|  | Extension | 72 °C | 20 seconds |  |
| TCIM | Initial Denaturation | 95 °C | 60 second | 1 |
|  | Denaturation | 95 °C | 15 seconds | **39** |
|  | **Annealing** | **60.7 °C** | **60 seconds** |  |
|  | Extension | 72 °C | 20 seconds |  |
| TFAP2C | Initial Denaturation | 95 °C | 60 second | 1 |
|  | Denaturation | 95 °C | 15 seconds | **39** |
|  | **Annealing** | **65.7 °C** | **60 seconds** |  |
|  | Extension | 72 °C | 20 seconds |  |
| TPM2 | Initial Denaturation | 95 °C | 60 second | 1 |
|  | Denaturation | 95 °C | 15 seconds | **39** |
|  | **Annealing** | **57 °C** | **60 seconds** |  |
|  | Extension | 72 °C | 20 seconds |  |
| TRAF6 | Initial Denaturation | 95 °C | 60 second | 1 |
|  | Denaturation | 95 °C | 15 seconds | **39** |
|  | **Annealing** | **57 °C** | **60 seconds** |  |
|  | Extension | 72 °C | 20 seconds |  |
| YPEL2 | Initial Denaturation | 95 °C | 60 second | 1 |
|  | Denaturation | 95 °C | 15 seconds | **39** |
|  | **Annealing** | **62 °C** | **60 seconds** |  |
|  | Extension | 72 °C | 20 seconds |  |
